# A Blood‐Derived Double‐Network Hydrogel with Robust Wet Adhesion for Keratinized Mucosa Regeneration via Neutrophil Phenotype Reprogramming and Mechanophysical Niche Modulation

**DOI:** 10.1002/advs.76188

**Published:** 2026-06-18

**Authors:** Sicong Ren, Xinting Yang, Jingxia Chen, Yihan Wang, Jian Feng, Jing Zhou, Jiaxin Luo, Jingjie Zhai, Quan Lin, Yanmin Zhou

**Affiliations:** ^1^ Jilin Provincial Key Laboratory of Tooth Development and Bone Remodeling Hospital of Stomatology Jilin University Changchun P. R. China; ^2^ Department of Stomatology Center for Regeneration and Aging Medicine the Fourth Affiliated Hospital of School of Medicine and International School of Medicine International Institutes of Medicine Zhejiang University Yiwu P. R. China; ^3^ State Key Laboratory of Supramolecular Structure and Materials College of Chemistry Jilin University Changchun P. R. China; ^4^ School of Stomatology Jilin University Changchun P. R. China

**Keywords:** autologous platelet concentrates, gingival fibroblasts, keratinized mucosa regeneration, neutrophils

## Abstract

Reconstruction of keratinized mucosa (KM) with sufficient dimensions is critical for long‐term periodontal and peri‐implant health. However, existing biomaterials struggle to recapitulate the complex biophysical and biochemical microenvironment of KM while achieving stable adhesion and integration in the wet and mechanically dynamic oral cavity. Here, we design a photocrosslinkable double‐network hydrogel composed of methacrylated platelet‐rich fibrin (iPRF‐MA), N‐hydroxysuccinimide‐functionalized alginate (Alg‐NHS), and luteolin‐loaded epigallocatechin gallate microspheres (Lut@EGCG) to enable KM regeneration in the challenging oral environment through dual microenvironmental modulation. At the material level, the covalent network from iPRF‐MA and the supramolecular network based on Alg‐NHS work synergistically, resulting in strong wet tissue adhesion and high fatigue resistance, which collectively prevent hydrogel dislodgement under oral dynamic stresses. Biochemically, the hydrogel enables sustained release of growth factors and EGCG, synergistically enhancing angiogenesis and immune regulation, while also redirecting neutrophil phenotype toward a phagocytic state for specific antibacterial activity. Biophysically, the hydrogel provides gingival fibroblasts with a mechanically instructive microenvironment that activates mechanotransduction signaling and accelerates extracellular matrix remodeling. In vivo experiments confirm outstanding KM regeneration following treatment with the double‐network hydrogel. This study demonstrates a microenvironment‐targeting strategy for KM reconstruction through rational hydrogel design, offering a therapeutic platform for functional KM regeneration.

## Graphical abstract

The iPRF‐MA/Alg‐NHS/Lut@EGCG (PGAE) double‐network hydrogel achieves durable wet adhesion in the oral environment. It simultaneously reprograms neutrophils from NETosis to phagocytosis for biofilm clearance, and activates the FAK‐RhoA‐YAP mechanotransduction pathway in gingival fibroblasts to drive extracellular matrix remodeling, thereby promoting functional keratinized mucosa regeneration.

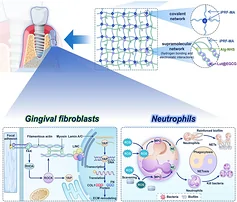

## 1 Introduction

Dental implant therapy represents the preferred treatment for partial edentulism [1, 2]. However, tooth loss is frequently accompanied by degradation of periodontal support, often resulting in keratinized mucosa (KM) that is inadequate in both quality and quantity [3, 4]. Since sufficient peri‑implant KM is essential for effective plaque control, marginal bone stability, and long‑term prosthetic success, KM augmentation remains a critical clinical procedure [5, 6]. Current augmentation techniques predominantly utilize autologous tissue grafts or commercial collagen membranes [7, 8]. Unfortunately, autografts are constrained by tissue availability and carry risks of postoperative pain, swelling, and donor‑site morbidity [9]. Collagen membranes, though effective, are costly and require technically sensitive handling, limiting their widespread use [10]. Therefore, improving KM augmentation strategies constitutes an important research direction in implant dentistry.

Autologous platelet concentrates (APCs) offer considerable potential in this context [11]. As autologous biological preparations, APCs contain a spectrum of endogenous growth factors at physiological ratios, ensuring excellent biocompatibility and eliminating immunogenic concerns [12]. Their clinical accessibility and proven performance in oral soft tissue repair make them attractive candidates for KM regeneration [13]. Nevertheless, two major limitations impede their practical application. First, conventional APCs exhibit uncontrollable gelation and poor wet tissue adhesion. Their pre‐gelled consistency hinders conformal adaptation to irregular wound surfaces, often necessitating suturing—a difficult task in the confined oral space. Moreover, salivary flow, dietary liquids, and oral movements collectively challenge material retention [11, 14]. Second, the inability to recreate the mechanical niche results in a failure to stimulate the mechanosensing pathways in gingival fibroblasts (GFs) [15]. GFs are pivotal orchestrators of KM regeneration, governing the synthesis and remodeling of the lamina propria's extracellular matrix (ECM) [16]. The mechanical microenvironment serves as a fundamental instructor of cell fate, and in KM—a tissue subjected to significant masticatory stress—GFs are uniquely responsive to mechanical cues. They critically mediate the process of mechanotransduction, converting physical stimuli into biochemical signals for ECM production [17, 18]. This underscores that the biophysical properties of their microenvironment, including substrate adhesion and stiffness, are essential regulators of GFs' function. However, conventional APCs possess suboptimal mechanical stiffness and fail to provide the necessary biophysical cues to GFs [19]. Hence, a functional upgrade of APCs that enhances wet adhesion and provides precisely tailored macroscopic and microscopic mechanical properties, while preserving innate regenerative cues, is indispensable for successful KM regeneration.

As biocompatible hydrogel systems, APCs must meet two critical requirements to achieve long‐term retention at KM defect sites: (1) strong adhesion to moist tissues and low intrinsic swelling, which helps minimize water diffusion and counteract the weakening effect of swelling stress on adhesion; and (2) high fatigue resistance to prevent dislodgement in the highly dynamic oral environment [20]. Moreover, the highly irregular morphology of mucosal defects complicates the precise fitting of pre‐formed hydrogels. A promising approach to overcome these challenges is to develop an in situ‐curing hydrogel that can be applied directly to the defect and, upon curing, exhibits robust adhesion, low swelling, and fatigue‐resistant properties. Achieving strong adhesion in wet environments hinges on the formation of rapid and stable covalent bonds with tissue surfaces. N‐hydroxysuccinimide (NHS) is commonly employed in the design of bioadhesives [21], because NHS‐activated ester groups can rapidly react with primary amines on tissue surfaces to form stable amide bonds, thereby enabling rapid covalent tissue adhesion [22, 23]. Alginate (Alg), which is naturally rich in carboxyl groups, can be functionalized with NHS ester groups to obtain Alg‐NHS, thereby providing activated carboxyl groups for tissue adhesion [24]. Therefore, building on our previously developed photo‐responsive APCs system—methacrylated platelet‐rich fibrin (iPRF‐MA) [11]—we sought to incorporate Alg‐NHS to construct a photocrosslinkable double‐network hydrogel with superior wet tissue adhesion. The abundant hydrogen bonds within this network contribute to low swelling behavior, while the double‐network architecture enhances fatigue resistance on the macroscopic level. At the microscopic scale, this structure increases the stiffness of the APCs, thereby providing more favorable biophysical cues to adherent GFs.

Inflammation and infection further complicate KM regeneration. Elevated inflammatory mediators at KM‑deficient sites exacerbate local immune responses and impede healing [25]. Conventional APCs lack anti‑inflammatory functionality, and their fibrin‑rich structure may even support microbial colonization, increasing biofilm risk [26]. Current antimicrobials often indiscriminately disrupt the oral microbiome, undermining ecological balance. As first responders to infection, neutrophils represent the primary defense against bacterial invasion and dissemination [27]. These cells possess a potent antimicrobial arsenal, with phagocytosis being their primary bactericidal mechanism [28]. However, in the oral cavity, all surfaces are coated with an acquired pellicle to which bacteria adhere and form biofilms. These consolidated biofilms resist phagocytosis, which in turn triggers the release of neutrophil extracellular traps (NETs) [29]. Notably, at KM defects characterized by a reactive oxygen species (ROS)‐rich microenvironment, neutrophils readily shift from phagocytosis toward NETosis, forming extensive meshworks that integrate into the biofilm's extracellular polymeric substance (EPS), thereby reinforcing the “NETs barrier” and enhancing biofilm resilience [30]. Moreover, NETosis leads to the release of intracellular autoantigens and enzymes, which can further disrupt the oral mucosal epithelial barrier [31]. Therefore, reprogramming neutrophil responses from NETosis toward phagocytosis emerges as a promising and specific strategy for combating oral biofilm infections.

**SCHEME 1:**
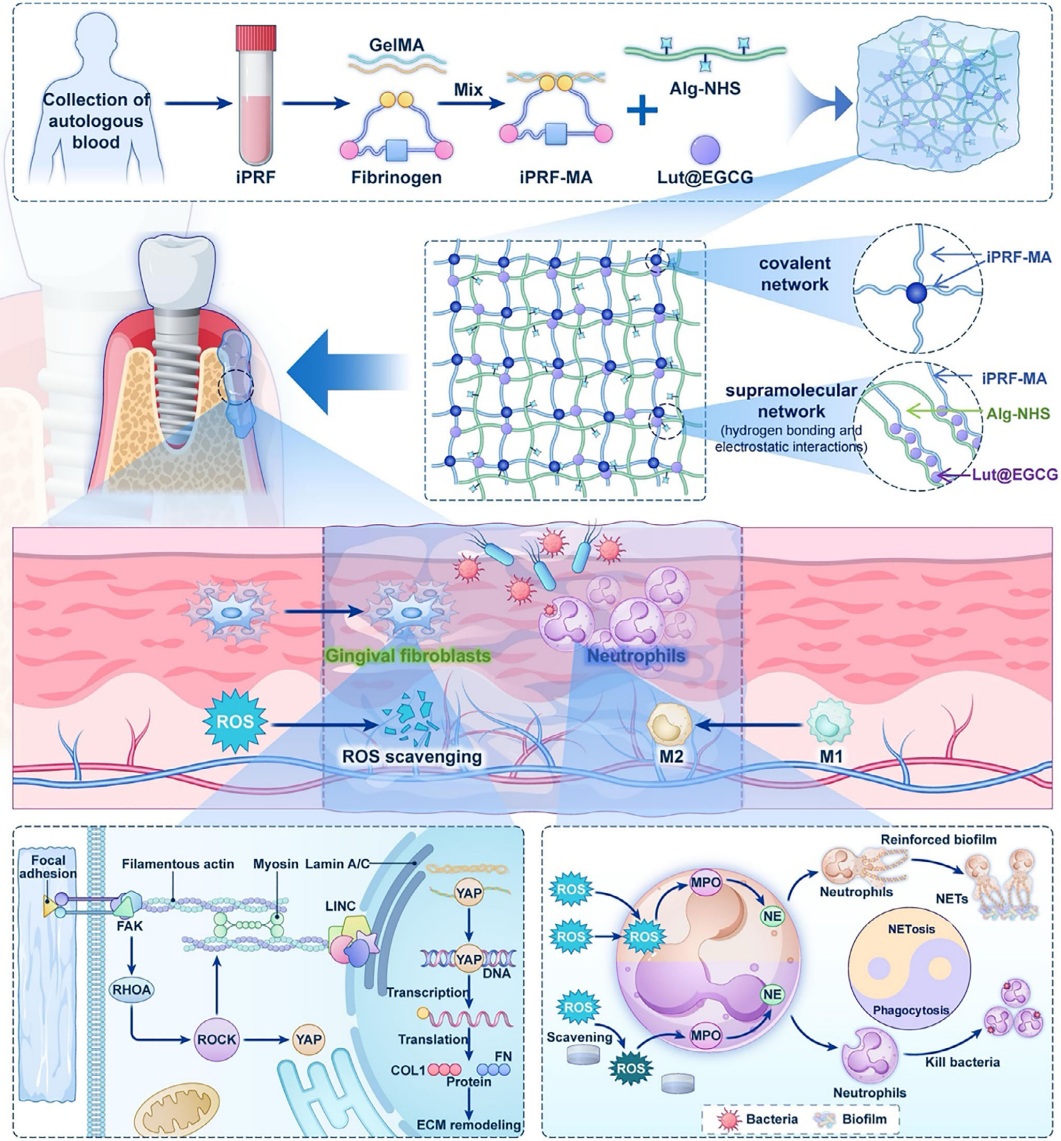
Schematic illustration of the blood‐derived double‐network hydrogel promoting keratinized mucosa regeneration through regulation of biophysical and biochemical cues.

Epigallocatechin gallate (EGCG), a natural polyphenol, exhibits both anti‑inflammatory and neutrophil‑reprogramming potential. However, its poor solubility and low bioavailability limit clinical utility [32, 33, 34]. In this study, to enable the stable and long‐term localization of EGCG at KM defects, we developed luteolin‐loaded EGCG microspheres (Lut@EGCG) and integrated them into a dual‐network hydrogel composed of iPRF‐MA and Alg‐NHS. The resulting iPRF‐MA/Alg‐NHS/Lut@EGCG (PGAE) hydrogel incorporates a covalent network formed through photo‐crosslinking of iPRF‐MA, along with a supramolecular network formed via hydrogen bonding and electrostatic interactions of Alg‐NHS. This design preserves the strong wet tissue adhesion capability of Alg‐NHS while establishing a photocrosslinkable dual‐network architecture. As a result, the hydrogel not only exhibits appropriate mechanical stiffness and tissue adhesion but also facilitates efficient energy dissipation, thereby preventing fatigue‐induced failure in the highly dynamic oral environment. On the biological front, this hydrogel system promotes vascularization at the KM defect by sustained release of endogenous growth factors. Simultaneously, the incorporated Lut@EGCG microspheres not only enhance the overall anti‐inflammatory capacity, but also selectively intercept the ROS–myeloperoxidase (MPO)/neutrophil elastase (NE) axis. This interception effectively reprograms neutrophil function by shifting the phenotype from NETosis to phagocytosis, thereby exerting a targeted antibacterial effect through immunomodulation. Furthermore, the hydrogel delivers essential biophysical cues to GFs, activating the focal adhesion kinase (FAK)–Ras homologous gene family member A (RhoA)–F‐actin–Yes‐associated protein (YAP) signaling pathway. This mechanotransduction cascade subsequently upregulates the synthesis of type I collagen (COL1) and fibronectin (FN), accelerating ECM remodeling and ultimately facilitating the regeneration of functional KM (Scheme 1).

## 2 Results and Discussion

### 2.1 Synthesis and Characterization of PGAE Hydrogels

**FIGURE 1:**
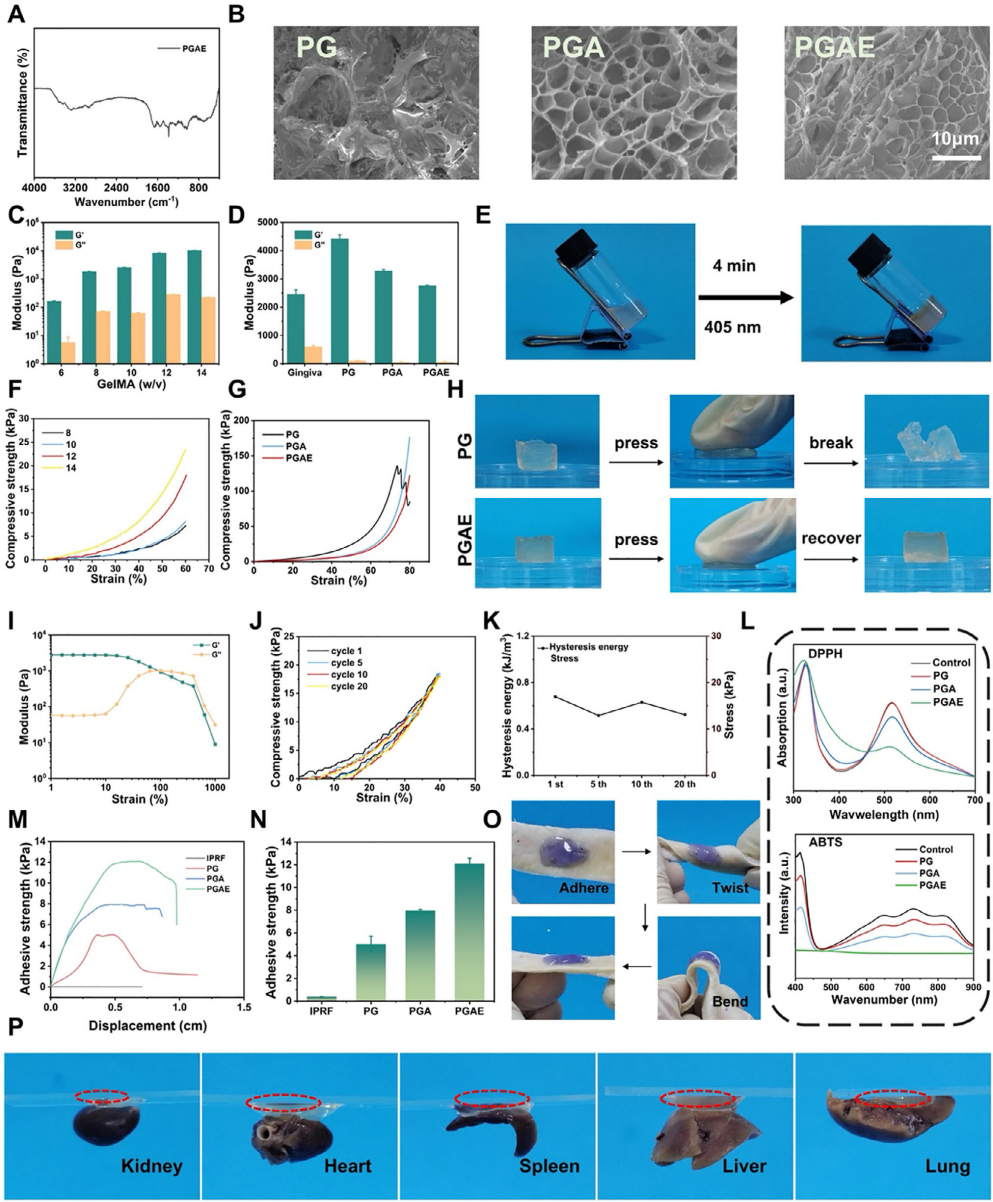
Synthesis and characterization of the PGAE hydrogel. (A) FTIR spectra of the PGAE hydrogel. (B) SEM images of different hydrogels. (C) Comparison of the modulus of PGAE hydrogels containing different ratios of GelMA. (D) Comparison of the modulus of natural KM, PG, PGA, and PGAE hydrogels. (E) Optical images of the gelation process of the PGAE hydrogel. (F) Comparison of the compressive properties of PGAE hydrogels containing different ratios of GelMA. (G) Comparison of the compressive properties of PG, PGA, and PGAE hydrogels. (H) Demonstration of the compressible performance of the PGAE hydrogel. (I) Strain sweep test results of the PGAE hydrogel. (J) Hysteresis loops of the PGAE hydrogel at the 1st, 5, 10, and 20th cycles. (K) Corresponding stress and hysteresis energy for each cycle. (L) DPPH and ABTS assay results of the hydrogels. (M) Adhesive strength‐displacement curves of different types of hydrogels. (N) Adhesive strength of different types of hydrogels. (O) Effect of twisting and bending deformations on the adhesion of PGAE hydrogel at hydrated interfaces. (P) Adhesion of the PGAE hydrogel to different wet biological surfaces.

EGCG, a plant‐derived polyphenol, exhibits potent anti‐inflammatory activity; however, its native form suffers from poor water solubility, short half‐life, low bioavailability, and limited contribution to hydrogel structural properties. To overcome these limitations, we co‐assembled luteolin (Lut) with EGCG into uniform microspheres (Lut@EGCG), enhancing dispersibility, hydrophilicity, and reinforcing both mechanical and anti‐inflammatory performance of the resulting hydrogel. The formation of Lut@EGCG microspheres was mainly driven by multiple non‐covalent interactions between luteolin and EGCG. EGCG contains abundant phenolic hydroxyl groups, catechol moieties, and galloyl groups, enabling extensive hydrogen‐bonding interactions. In our system, EGCG could bind to Gly through hydrogen bonding and thereby modulate EGCG‐induced molecular aggregation. In addition, luteolin contains phenolic hydroxyl groups, a carbonyl group, and a planar flavonoid aromatic skeleton, which can interact with EGCG through multiple hydrogen bonds. The aromatic domains of luteolin and EGCG may promote π–π stacking, while the relatively hydrophobic flavonoid backbone of luteolin contributes to hydrophobic aggregation in aqueous media. These interactions further facilitate the nucleation and stabilization of the spherical co‐assembled structures. Zeta potential measurements confirmed successful complexation between Lut and EGCG (Figure S1). SEM imaging and particle size analysis revealed Lut@EGCG microspheres of ≈200 nm with a narrow size distribution (Figures S2 and S3), indicating favorable compatibility and uniform dispersion within the hydrogel network. UV–vis spectroscopy displayed an absorption peak at 278 nm corresponding to the catechol groups, along with a broad band at 353 nm characteristic of luteolin, confirming successful incorporation (Figure S4). Subsequently, iPRF‐MA and Alg‐NHS were synthesized individually. In the presence of the photoinitiator lithium phenyl‐2,4,6‐trimethylbenzoylphosphinate (LAP), the iPRF‐MA/Alg‐NHS precursor solution was exposed to blue light (405 nm) for 4 min, forming a covalently crosslinked iPRF‐MA network interpenetrated with Alg‐NHS. Finally, Lut@EGCG was introduced, establishing dynamic hydrogen bonds with iPRF‐MA that enriched the crosslinking network and further enhanced the mechanical properties of the hydrogel. For clarity in subsequent sections, the following designations are used: iPRF‐MA as PG, iPRF‐MA/Alg‐NHS as PGA, and iPRF‐MA/Alg‐NHS/Lut@EGCG as PGAE. Unless otherwise stated, the PGAE formulation used in the subsequent mechanical, fatigue, wet adhesion, and biological experiments contained 1% Alg‐NHS, 0.4 mg/mL Lut@EGCG, and 0.02% LAP. Fourier transform infrared (FTIR) spectroscopy verified the successful synthesis of PGAE hydrogel, with characteristic peaks observed at 1672 cm^−^^1^ (C = C of GelMA), 1650 cm^−^^1^ (COOH of Alg), and 1384 cm^−^^1^ (phenolic hydroxyl of Lut@EGCG) (Figure 1A). SEM imaging (Figure 1B) indicated that both PGAE and PGA hydrogels exhibited smaller pore sizes compared to PG, attributable to increased crosslinking density imparted by non‐covalent interactions such as hydrogen bonding and electrostatic forces. To assess the photoresponsive behavior, the gelation process of PGAE was evaluated. The hydrogel maintained an injectable liquid state at room temperature and underwent rapid sol–gel transition under 405 nm light exposure, enabling controlled application to tissue defects (Figure 1E).

An ideal KM repair material should exhibit mechanical properties comparable to those of native tissue to ensure seamless integration at the defect site. Our measurements indicated that the storage modulus of natural KM is approximately 2.4 kPa. Accordingly, we modulated the concentration of GelMA—the primary structural component of the hydrogel—to align the modulus of the PGAE hydrogel with that of the target tissue. As illustrated in Figure 1C,D, a GelMA concentration of 10% yielded a hydrogel modulus most closely matching that of KM, and this formulation was adopted in all subsequent experiments. Storage modulus (G′) was selected as the primary mechanical matching parameter because it reflects the elastic energy‐storage capacity of hydrated hydrogels under small‐amplitude oscillatory deformation and provides an effective estimate of the elastic stiffness sensed by cells and surrounding tissues. This parameter is particularly relevant for the present hydrogel system, as the PGAE hydrogel was designed not only to cover KM defects but also to provide gingival fibroblasts with a mechanically instructive microenvironment. Therefore, matching the G′ value of PGAE with that of native KM was used as a key criterion for optimizing the GelMA content. In addition, the swelling behavior of the PGAE hydrogel was evaluated in PBS at 37°C to assess its adaptability to the moist oral environment. As shown in Figure S6, the PGAE hydrogel exhibited a limited swelling ratio after 12 h, which may be attributed to the dense double‐network structure formed by photocrosslinked iPRF‐MA, Alg‐NHS, and Lut@EGCG‐mediated non‐covalent interactions. This restricted swelling behavior may help reduce water‐induced weakening of tissue adhesion and support hydrogel retention under continuous salivary flushing. PGAE hydrogels were exposed to 405 nm light for different durations to evaluate changes in modulus. As shown in Figure S7, the modulus increased with prolonged light exposure and reached a relatively stable level after 4 min. We further evaluated the rheological behavior of the PGAE hydrogel. As shown in Figure S5, both the storage modulus (G′) and loss modulus (G″) remained stable throughout the testing period, indicating dominant elastic solid characteristics. Strain sweep tests revealed that G″ only exceeded G′ at strains above 100%, reflecting the hydrogel's structural collapse from a gel to sol state and confirming its considerable toughness (Figure 1I). Compression testing demonstrated that the compressibility of the PGAE hydrogel increased with higher GelMA content (Figure 1F). Notably, the PGAE hydrogel exhibited significantly enhanced compressibility relative to the PG hydrogel. Under 70% strain, the PG hydrogel fractured, whereas the PGAE hydrogel maintained its structural integrity (Figure 1G). Visual evidence of its deformation recovery further attests to the excellent toughness of the material (Figure 1H). Beyond adequate modulus and strength, the ability of a hydrogel to recover after repeated compression is essential for withstanding oral physiological loads. Thus, fatigue resistance is a critical design criterion for KM repair materials. Cyclic compression tests (Figure 1J) showed that the PGAE hydrogel could undergo 20 loading–unloading cycles at 40% strain with immediate shape recovery. The consistent hysteresis loops observed across all cycles indicated minimal plastic deformation and excellent fatigue resistance. Corresponding stress and hysteresis energy analysis further confirmed stable mechanical behavior over the cycles (Figure 1K). Collectively, these results demonstrate that the hydrogen bonding between iPRF‐MA and Lut@EGCG, combined with electrostatic interactions between iPRF‐MA and Alg‐NHS, imparts high stiffness, efficient energy dissipation, and robust elasticity to the hydrogel. This multi‐network design ensures high toughness and fatigue resistance, effectively preventing hydrogel dislodgement in the highly dynamic oral environment.

Beyond forming strong adhesion to tissues in dry conditions, an ideal KM repair material must maintain robust interfacial bonding in the presence of water—a key challenge in the wet oral environment. To evaluate the wet tissue adhesion capability of the hydrogel, we applied a thin layer of water onto porcine skin using a pipette, added the hydrogel precursor to the hydrated surface, and allowed it to cure for shear adhesion testing. As shown in Figure 1O, the PGAE hydrogel maintained firm adhesion to the tissue even at the wet interface, with no detachment under torsional or bending deformation. We further quantified the wet adhesion strength across different hydrogel formulations. While iPRF exhibited negligible tissue adhesion in the presence of interfacial water, both PG and PGA showed progressively improved adhesive strength, with PGAE demonstrating the highest value (Figure 1N). This enhancement is attributed to Alg‐NHS, which rapidly reacts with amino groups on the tissue surface to form stable amide bonds, providing reactive sites for durable adhesion. Additionally, the catechol groups present in Lut@EGCG further reinforce interfacial binding via hydrogen bonding with the tissue. The dense hydrogen‐bonding network within the hydrogel also helps to minimize water diffusion and swelling‐induced weakening of adhesion. The broad plateau in the adhesion strength–displacement curve of PGAE hydrogel, which surpasses that of PGA (Figure 1M), further confirms the critical role of catechol groups from Lut@EGCG in enhancing wet adhesion. Moreover, the PGAE hydrogel exhibited effective adhesion to various moist biological surfaces (Figure 1P). Cell proliferation assays revealed that the PGAE hydrogel not only preserved the bioactivity of iPRF but also significantly enhanced its ability to promote cell growth (Figure S8).

### 2.2 The Role of PGAE Hydrogel in Modulating the Biochemical Microenvironment for Keratinized Mucosa Regeneration

**FIGURE 2:**
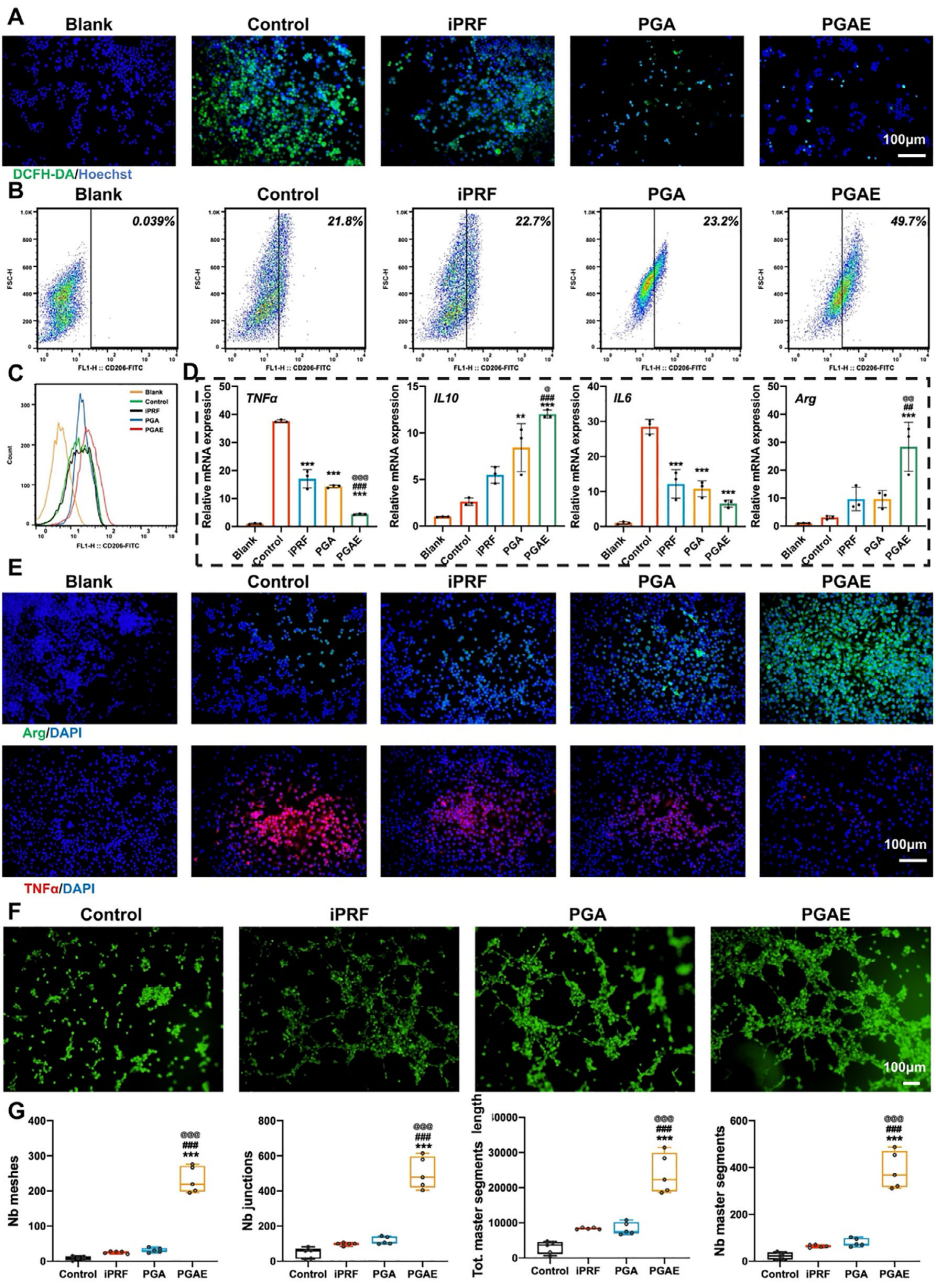
The ability of PGAE hydrogel to regulate the biochemical microenvironment for keratinized mucosa regeneration. (A) Scavenging effect of the PGAE hydrogel on macrophage intracellular ROS. (B) Proportion of M2 macrophages after treatment with different hydrogels. (C) Overlaid histograms of the flow cytometry results. (D) Expression of inflammation‐related genes in macrophages after treatment with different hydrogels. (E) Fluorescence staining results of inflammation‐related proteins in macrophages. (F) Fluorescence images from the tube formation assay. (G) Quantitative analysis of the tube formation assay, including the number of meshes, number of junctions, total master segments length, and number of master segments (Compared to the Control group, * indicates *p* < 0.05, ** indicates *p* < 0.01, and *** indicates *p* < 0.001; compared to the iPRF group, ^#^ indicates *p* < 0.05, ^##^ indicates *p* < 0.01, and ^###^ indicates *p* < 0.001; compared to the PGA group, ^@^ indicates *p* < 0.05, ^@@^ indicates *p* < 0.01, and ^@@@^ indicates *p* < 0.001).

The regeneration of KM often occurs within a chronically inflamed microenvironment, driven by the persistent presence of oral microbes and continual mechanical stimulation from physiological activities. The loss of the mucosal barrier further exacerbates these adverse conditions, which are characterized by excessive ROS production, dysregulated immune responses, and impaired vascularization. To evaluate the ability of the biomimetic hydrogel to modulate this regenerative microenvironment, we systematically assessed its antioxidant, immunomodulatory, and pro‐angiogenic properties. DPPH and ABTS assays confirmed the hydrogel's potent antioxidant activity (Figure 1L). In a cellular model of oxidative stress induced by lipopolysaccharide (LPS) in RAW264.7 macrophages, treatment with the PGAE hydrogel significantly reduced intracellular ROS levels, as indicated by diminished fluorescence intensity (Figure 2A). Macrophages, as key regulators of inflammation, can polarize into pro‐inflammatory M1 or pro‐regenerative M2 phenotypes. In defects with KM deficiency, macrophages often remain arrested in the M1 state, sustaining inflammation and impairing healing. Flow cytometry and Real‐time quantitative polymerase chain reaction (RT‐qPCR) analyses revealed that LPS‐stimulated macrophages exhibited decreased expression of the M2 marker CD206 and increased M1 markers (TNF‐α and IL‐6). In contrast, PGAE treatment promoted a shift toward the M2 phenotype, with elevated expression of IL‐10 and arginase (Arg) (Figure 2B–D). These findings were corroborated at the protein level by immunofluorescence staining, which showed decreased TNF‐α and iNOS alongside increased IL‐10 and Arg in the PGAE group (Figure 2E and Figures S10 and S11). We further investigated the pro‐angiogenic capacity of PGAE using human umbilical vein endothelial cells (HUVECs). Scratch wound and Transwell migration assays demonstrated that PGAE enhanced HUVEC migration (Figure S9). In tube formation assays, PGAE‐treated cells developed more extensive and defined tubular networks, indicating strong pro‐angiogenic potential (Figure 2F,G). In summary, the PGAE hydrogel effectively reshapes the local immune microenvironment and stimulates vascular network formation, thereby improving oxygen and nutrient supply to create favorable conditions for KM regeneration.

### 2.3 Reprogramming Neutrophil Phenotypes for Specific Antibiofilm Action with PGAE Hydrogels

**FIGURE 3:**
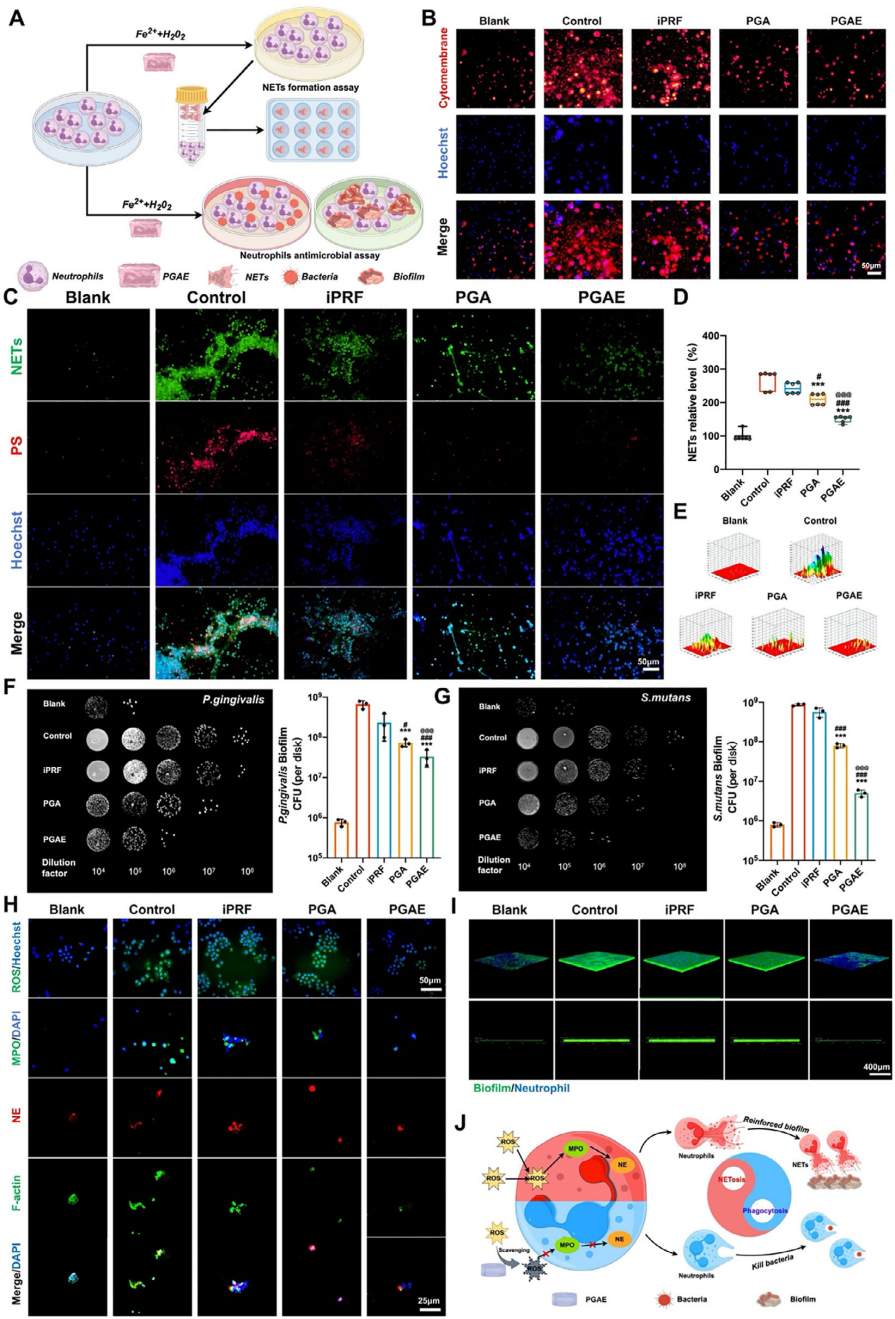
Capability and mechanism of the PGAE hydrogel in reprogramming neutrophil phenotypes. (A) Schematic diagram of the neutrophil‐related assays. (B) Cell membrane staining of neutrophils after different treatments. (C) NETs and PS staining results of neutrophils after different treatments. (D) Quantitative analysis of NETs levels in neutrophils after different treatments. (E) Quantitative analysis of PS fluorescence intensity. (F) Colony images and CFU counts of *P. gingivalis* after treatment with neutrophils that were preconditioned by different hydrogels. (G) Colony images and CFU counts of *S. mutans* after treatment with neutrophils that were preconditioned by different hydrogels. (H) Expression levels of ROS, MPO, NE, and F‐actin in neutrophils after treatment with different hydrogels. (I) Fluorescence images of composite biofilms after treatment with neutrophils. (J) Mechanistic schematic of hydrogel‐mediated functional reversal of neutrophils (Compared to the Control group, * indicates *p* < 0.05, ** indicates *p* < 0.01, and *** indicates *p* < 0.001; compared to the iPRF group, ^#^ indicates *p* < 0.05, ^##^ indicates *p* < 0.01, and ^###^ indicates *p* < 0.001; compared to the PGA group, ^@^ indicates *p* < 0.05, ^@@^ indicates *p* < 0.01, and ^@@@^ indicates *p* < 0.001).

The loss of the epithelial barrier at KM defect sites facilitates bacterial colonization and the development of stubborn biofilm infections. This infection, in turn, critically impedes the regeneration process. Therefore, effective elimination of biofilm is a fundamental prerequisite for successful KM regeneration. Neutrophils serve as essential antibacterial effectors in the human immune system and constitute the first line of defense against bacterial invasion and infection [35]. They efficiently eliminate bacteria at oral wound sites without disrupting the commensal microbiota, making them an attractive target for achieving pathogen‐specific antimicrobial activity while maintaining oral ecological balance. Recent evidence suggests that under conditions of high oxidative stress at KM defect sites, excessive ROS can redirect neutrophil fate from phagocytosis—a primary bactericidal mechanism—toward a form of cell death known as NETosis, leading to the release of NETs. In the context of oral biofilm infection, NETs become embedded within the EPS of biofilms, reinforcing the structural barrier and impeding biofilm dispersal. Furthermore, excessive NETs formation exacerbates mucosal barrier damage, perpetuating a cycle of inflammation and infection. Therefore, reprogramming neutrophils from NETosis toward a phagocytic phenotype represents a promising strategy for resolving oral biofilm infections. As illustrated in Figure 3A, we mimicked the high oxidative stress microenvironment of KM defect sites by treating neutrophils with FeSO_4_ and H_2_O_2_ and evaluated the effect of different treatments on neutrophil behavior. Since membrane integrity is essential for phagocytosis and is disrupted during NETosis, we used the lipophilic DiI probe to label the phospholipid bilayer. Under high ROS conditions, neutrophil membranes exhibited severe disruption, which was effectively rescued by PGAE treatment, indicating preserved membrane integrity and functional competence (Figure 3B). We further assessed NETs formation under different culture conditions. Robust extracellular green fluorescent structures, indicative of active NETs generation, were observed in the control group under high ROS. In contrast, PGAE treatment significantly suppressed NETs release. To further clarify neutrophil status, we examined phosphatidylserine (PS) externalization—a hallmark of cell death. Control neutrophils showed pronounced PS exposure and nuclear fragmentation, whereas the PGAE group displayed minimal PS exposure and maintained nuclear integrity (Figure 3C–E). Quantitative analysis of NETs collected from each group confirmed that PGAE resulted in the lowest NETs levels (Figure 3D). Collectively, these findings demonstrate that high ROS levels drive neutrophils toward NETosis, while PGAE preserves membrane integrity, suppresses NETs formation, and redirects neutrophils toward a phagocytic phenotype.

We then evaluated the antibacterial performance of neutrophils under different treatment conditions. To this end, we first established single‐species biofilms of two common oral bacteria, *Porphyromonas gingivalis* and *Streptococcus mutans*, and investigated the ability of differently treated neutrophils to combat these biofilms. After co‐culturing neutrophils with pre‐formed biofilms, the remaining biofilm biomass was quantified using crystal violet staining. As shown in Figure S12, untreated neutrophils effectively cleared the biofilms, whereas those exposed to high oxidative stress lost this antibacterial capacity. In contrast, neutrophils treated with the PGAE hydrogel exhibited significantly enhanced bacterial phagocytosis and effectively disrupted the biofilms. These observations were further supported by colony‐forming unit (CFU) assays, which confirmed that the PGAE hydrogel restored the bactericidal function of neutrophils under oxidative stress (Figure 3F,G). Furthermore, we employed confocal laser scanning microscopy to visualize neutrophil activity against multispecies oral biofilms. As depicted in Figure 3I, untreated neutrophils induced structural damage and dispersal of the biofilm architecture. Under high oxidative stress, however, neutrophils lost this disruptive capacity and instead released NETs that adhered to the biofilm surface, reinforcing the barrier function of the biofilm and resulting in a dense, intact bacterial structure. Notably, treatment with the PGAE hydrogel restored the phagocytic ability of neutrophils, leading to pronounced structural degradation of the biofilm (Figure S13).

Encouraged by these findings, we further investigated the mechanism by which the PGAE hydrogel reprograms neutrophil function using immunofluorescence staining. The generation of ROS, together with upregulated expression and nuclear translocation of NE and MPO, is a key event in NETosis [30]. We therefore performed immunofluorescence staining of ROS, MPO, and NE in neutrophils to elucidate the underlying mechanism. Results revealed a sharp increase in intracellular and extracellular ROS levels in neutrophils under high oxidative stress compared to the Blank group, whereas PGAE treatment significantly suppressed ROS production. Staining of MPO and NE showed markedly enhanced expression and distinct nuclear migration in neutrophils under oxidative stress, as visualized by cytoskeletal counterstaining. Importantly, PGAE treatment not only reduced MPO and NE expression but also effectively prevented their nuclear translocation (Figure 3H and Figure S14). These results demonstrate that the PGAE hydrogel acts as an effective immunomodulatory regulator for neutrophils, redirecting their function from NETosis to phagocytosis by blocking the ROS–MPO/NE axis under high ROS conditions (Figure 3J).

### 2.4 Dual Modulation of the Bio‐Physicochemical Milieu of Gingival Fibroblasts by PGAE Hydrogels

**FIGURE 4:**
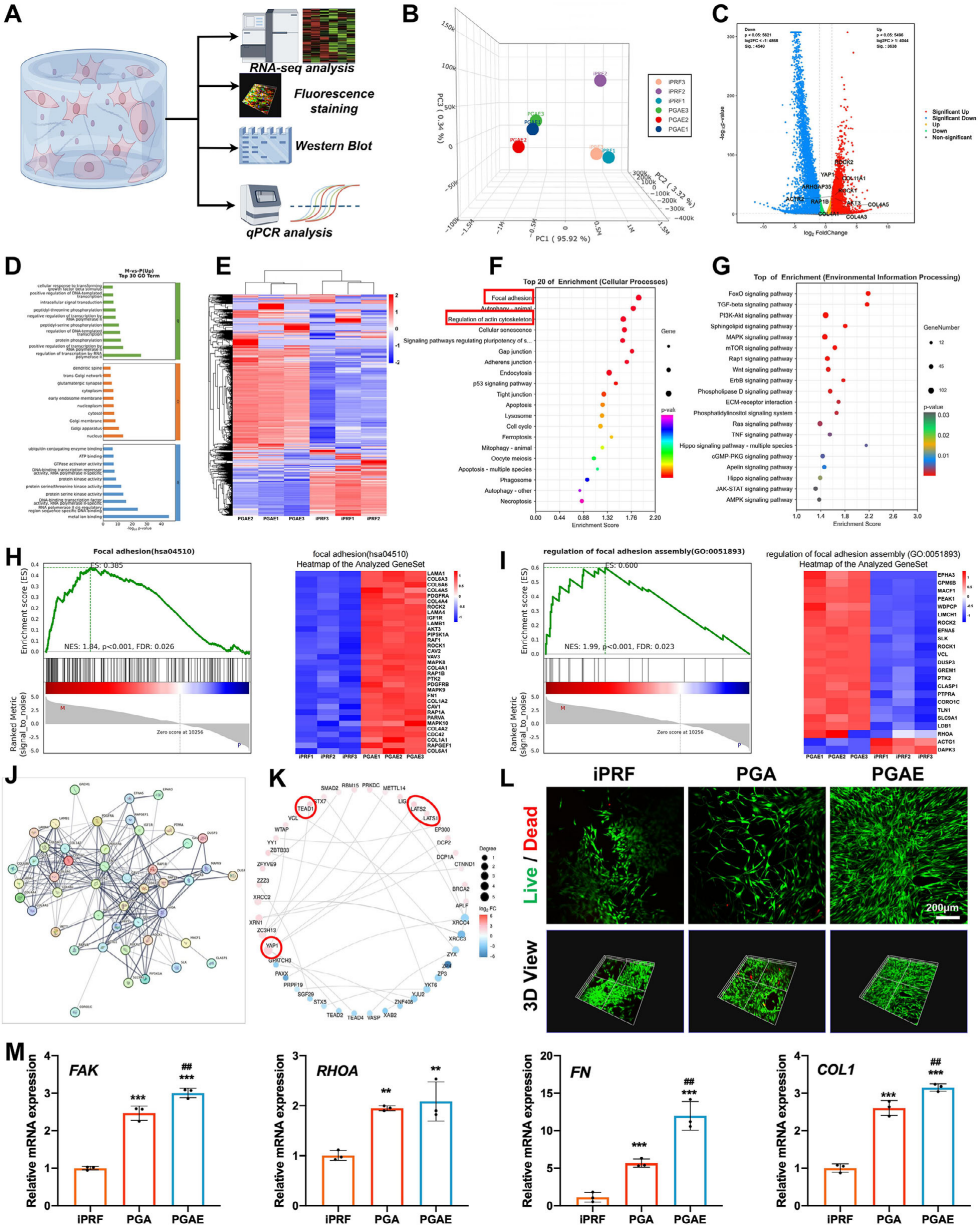
Regulatory effects of the PGAE hydrogel on the mechanical microenvironment of gingival fibroblasts. (A) Schematic diagram of the 3D culture model and related assays. (B) PCA of the RNA‐seq data. (C) Volcano plot of the DEGs. (D) GO enrichment analysis results. (E) Heatmap of the DEGs. (F) Top 20 upregulated pathways in “cellular process”. (G) Top 20 upregulated pathways in “environmental information process”. (H) GSEA and heatmap analysis for the FA signaling pathway. (I) GSEA and heatmap analysis for the “Regulation of FA assembly” signaling pathway. (J) PPI network for the FA pathway. (K) PPI network for the YAP signaling pathway. (L) Morphology and distribution of GFs cultured on different hydrogels. (M) Gene expression levels of FAK, RhoA, FN, and COL1 in GFs cultured on different hydrogels (Compared to the iPRF group, * indicates *p* < 0.05, ** indicates *p* < 0.01, and *** indicates *p* < 0.001; compared to the PGA group, ^#^ indicates *p* < 0.05, ^##^ indicates *p* < 0.01, and ^###^ indicates *p* < 0.001).

Given that the PGAE hydrogel exhibits a 100‐fold greater mechanical strength than conventional APCs, we further investigated whether this macroscale enhancement could translate into additional biophysical cues at the cellular level. To investigate the biological effects of the PGAE hydrogel, we first performed colony formation and wound healing assays, which demonstrated that the biochemical signals (e.g., growth factors) released from the PGAE hydrogel significantly enhanced the proliferation and migration capacities of GFs (Figure S15). To further evaluate the influence of the biophysical cues provided by the hydrogel matrix, GFs were encapsulated and cultured within the PGAE hydrogel under three‐dimensional conditions. RNA sequencing was employed to dissect the underlying molecular mechanisms (Figure 4A). Principal component analysis (PCA) confirmed good reproducibility among replicates (Figure 4B). Volcano plot analysis identified 3610 upregulated and 4502 downregulated differentially expressed genes (DEGs) in the PGAE group compared to the control (Figure 4C). The heatmap depicts the expression patterns of these DEGs (Figure 4E). Enrichment analyses of Gene Ontology (GO) and Kyoto Encyclopedia of Genes and Genomes (KEGG) pathways highlighted the top 20 terms in “cellular process” and “environmental information processing”. Notably, GFs cultured on PGAE showed significant upregulation of the focal adhesion (FA) and actin cytoskeleton regulation pathways (Figure 4D–G). Gene set enrichment analysis (GSEA) further supported the activation of FA‐related processes. Heatmap analysis further demonstrated a significant upregulation in the expression of genes encoding key components of the FA pathway (ROCK, RhoA, CAV1), as well as those critical for the synthesis of ECM essential for KM, such as COL1 and FN (Figure 4H,I). Protein–protein interaction (PPI) networks constructed using STRING revealed strong correlations among FA‐related molecules, and further analysis indicated significant upregulation of Hippo‐YAP pathway components (YAP, TEAD, LATS), underscoring the role of mechanotransduction in PGAE‐mediated GFs activation (Figure 4J,K).

Studies have shown that GFs are capable of sensing mechanical cues from their microenvironment, with FA serving as critical mechanosensing structures [16, 36, 37]. The core FA component, focal adhesion kinase (FAK), becomes activated under high mechanical stimulation and subsequently promotes nuclear translocation of YAP through RhoA and ROCK signaling [38]. Additionally, activated FAK facilitates nuclear pore opening via actin cytoskeleton reorganization, further enhancing YAP nuclear import [39, 40]. YAP, a mechanosensitive transcriptional regulator, translates mechanical signals into gene expression changes. Upon nuclear translocation in GFs, YAP binds to TEAD and drives the expression of key ECM remodeling proteins—such as COL1 and FN—that are essential for KM regeneration [41]. Based on these insights and our RNA‐seq findings, we hypothesize that—compared to conventional blood‐derived products—the PGAE hydrogel provides enhanced substrate stiffness and adhesion sites, leading to FAK activation. This in turn upregulates RhoA and ROCK activity, promotes cytoskeletal reorganization, and facilitates YAP nuclear accumulation, ultimately driving COL1 and FN expression, enhancing ECM synthesis, and accelerating KM regeneration.

**FIGURE 5:**
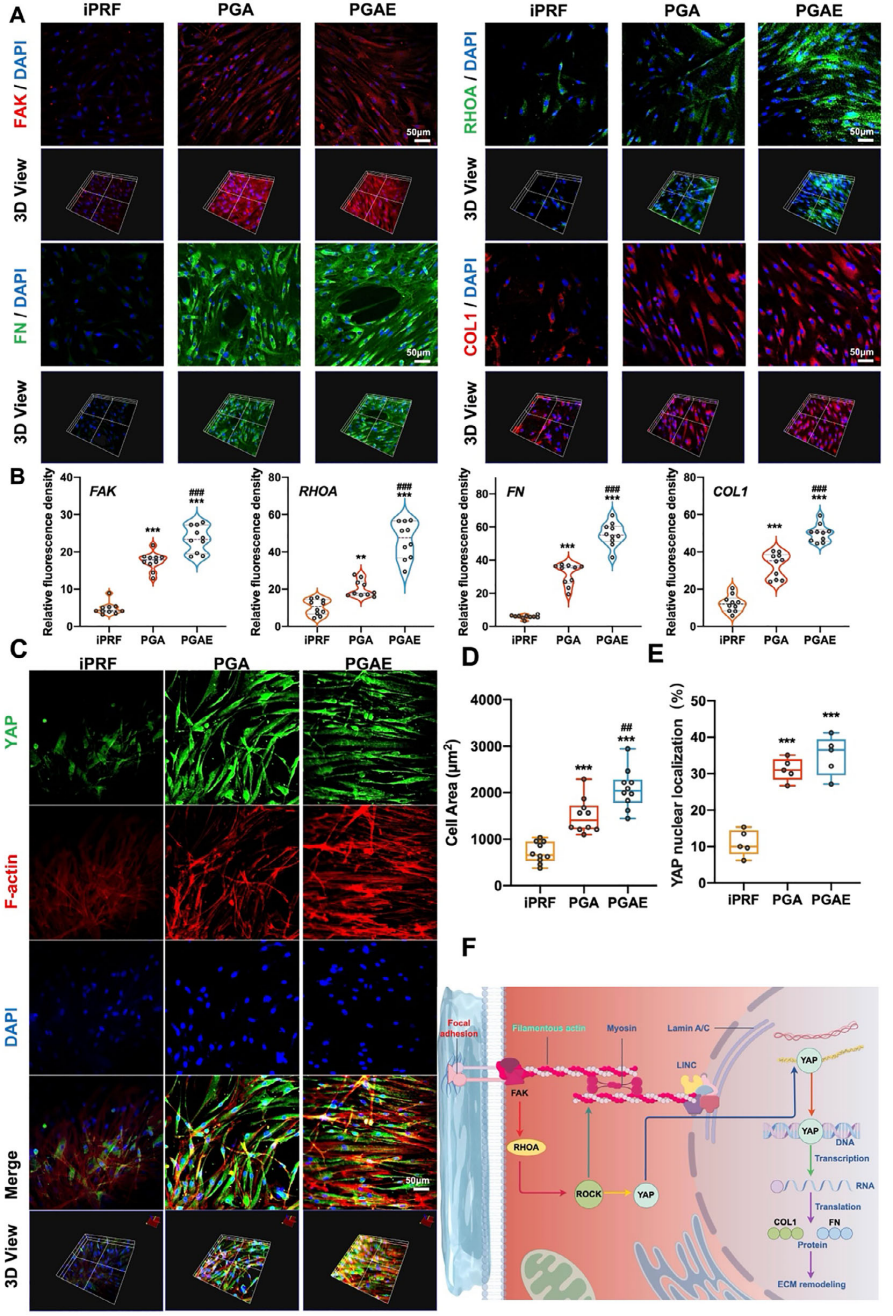
Activation of mechanical signaling pathways in gingival fibroblasts by the PGAE hydrogel. (A) Immunofluorescence staining of FAK, RhoA, FN, and COL1 in GFs cultured on different hydrogels. (B) Quantitative analysis of the fluorescence intensity for FAK, RhoA, FN, and COL1. (C) Immunofluorescence staining of YAP protein and F‐actin in GFs cultured on the hydrogels. (D) Quantitative analysis of cell spreading area. (E) Quantitative analysis of the nuclear translocation rate of YAP protein. (F) Mechanistic schematic illustrating how the PGAE hydrogel activates mechanical signal transduction in GFs and promotes ECM remodeling (Compared to the iPRF group, * indicates *p* < 0.05, ** indicates *p* < 0.01, and *** indicates *p* < 0.001; compared to the PGA group, ^#^ indicates *p* < 0.05, ^##^ indicates *p* < 0.01, and ^###^ indicates *p* < 0.001).

To validate the RNA‐seq results, we performed RT‑qPCR, immunofluorescence staining, and western blot analyses. Live/dead staining of GFs cultured in the materials revealed that the PGAE group exhibited the most pronounced pro‐proliferative effect, with cells displaying a more spread morphology (Figure 4L). RT‑qPCR confirmed significantly elevated expression of FAK, RhoA, COL1, and FN in the PGAE group compared to iPRF (Figure 4M). These findings were corroborated by immunofluorescence imaging and intensity analysis, indicating activation of mechanosignaling in GFs (Figure 5A,B). F‑actin staining showed more extended cytoskeletons and larger cell areas in GFs cultured in PGAE. YAP staining revealed distinct subcellular localization patterns in GFs: while YAP was predominantly localized in the cytoplasm of GFs cultured within iPRF, those encapsulated in the PGAE hydrogel exhibited markedly enhanced nuclear accumulation of YAP (Figure 5C–E). Subsequent Western blot analysis confirmed the protein expression levels of FAK, RhoA, COL1, and FN in treated cells. Notably, the PGAE group exhibited the highest expression of all four proteins. Furthermore, nuclear protein extraction and blotting revealed that the PGAE hydrogel also induced the most pronounced nuclear translocation of YAP in GFs, indicating strong activation of this mechanosensitive pathway (Figure S16). In summary, beyond delivering biochemical signals that promote GFs proliferation and migration, the PGAE hydrogel activates the FAK–RhoA–F‑actin–YAP signaling axis, stimulating the synthesis of COL1 and FN, enhancing ECM production, and ultimately supporting the regeneration of KM (Figure 5F).

### 2.5 The In Vivo Pro‐Regenerative Effect of PGAE Hydrogels on Keratinized Mucosa

**FIGURE 6:**
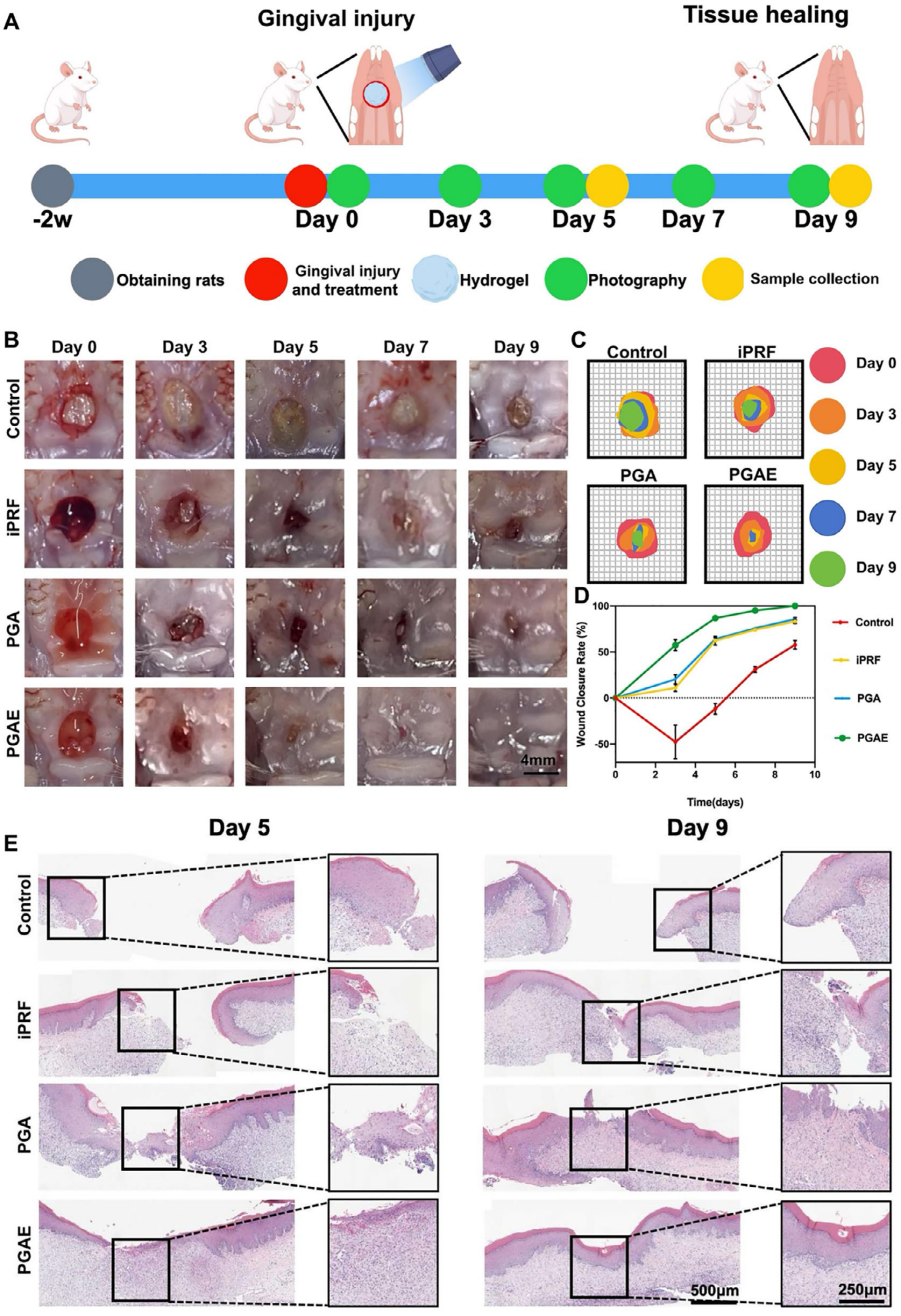
In vivo therapeutic efficacy of the PGAE hydrogel. (A) Schematic diagram of the establishment of the KM defect model and the subsequent treatment regimen. (B) Representative photographic images of the wound sites in different groups at the indicated time points. (C) Simulated diagrams depicting the wound area of the KM in different groups at corresponding time points. (D) Quantitative analysis of the wound healing rate in different groups. (E) Hematoxylin and eosin (H&E) staining results of the wound areas at different time points.

The palatal mucosa, a type of KM, is the preferred graft source in clinical practice for addressing peri‑implant KM deficiency due to its structural and functional similarity. To evaluate the therapeutic potential of the PGAE hydrogel for oral KM regeneration, we established a rat palatal mucosa defect model (Figure 6A). Wound healing was systematically monitored and photographed on postoperative days 0, 3, 5, 7, and 9, with quantitative analysis of the wound area. The PGAE‑treated group exhibited the most rapid wound closure at all time points, whereas the control group showed a tendency for wound expansion on days 3 and 5 (Figure 6B–D). Notably, the PGAE hydrogel could be injected into the defect and photocrosslinked in situ, enabling its stable retention at the wound site. As shown in Figure 6B, the hydrogel remained adhered to the tissue defect even on day 3. In addition, bacteria collected from the wound and surrounding tissue surfaces were quantified, revealing that the PGAE hydrogel possessed significantly stronger antibacterial efficacy in vivo compared to conventional APCs (Figure S17).

**FIGURE 7:**
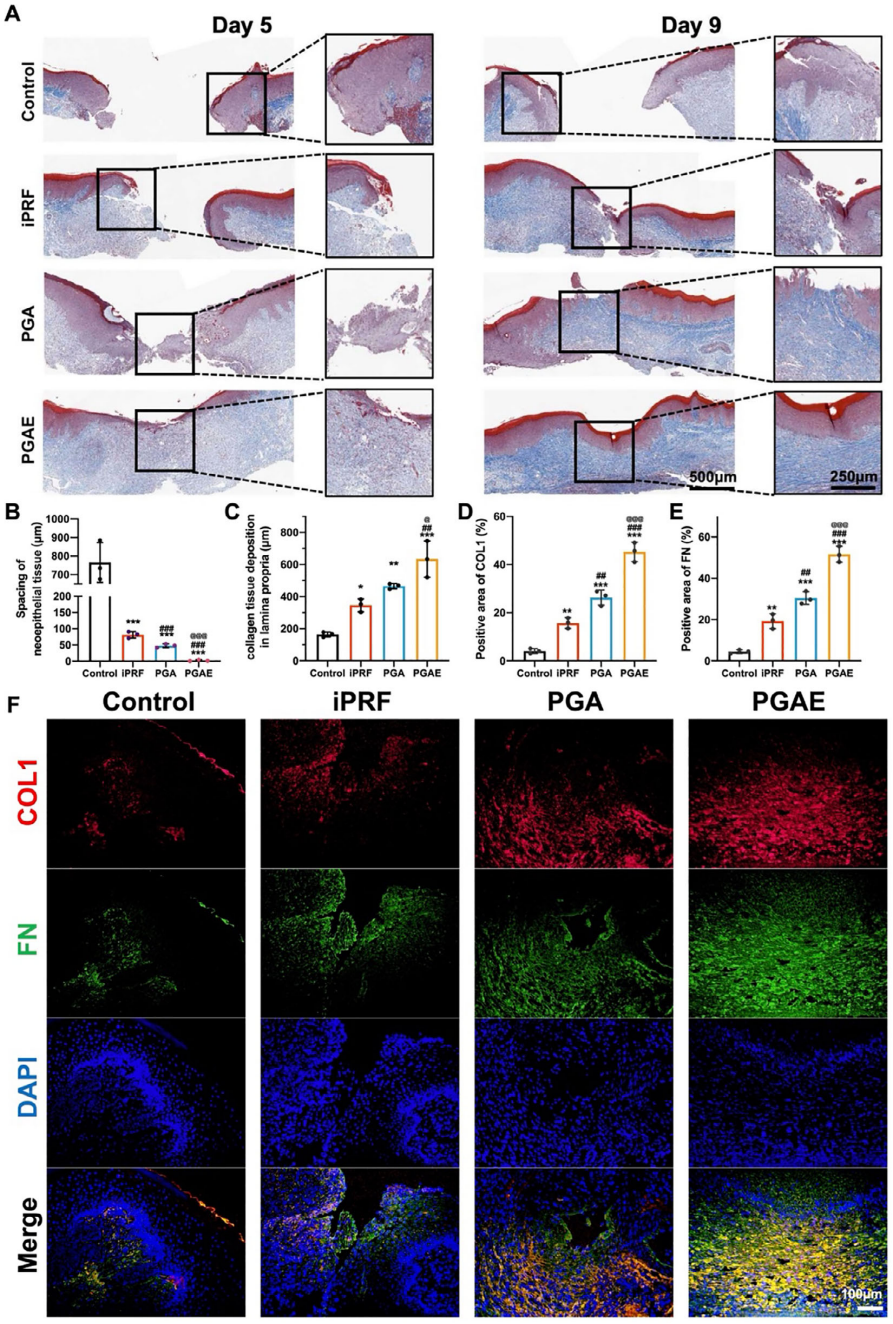
In vivo therapeutic effects of the PGAE hydrogel. (A) Masson's trichrome staining results at different time points. (B) Quantitative analysis of epithelial tissue spacing. (C) Quantitative analysis of collagen deposition in the lamina propria. (D) Quantitative analysis of COL1 expression levels in the newly formed tissue. (E) Quantitative analysis of FN expression levels in the newly formed tissue. (F) Immunofluorescence staining of COL1 and FN in the newly regenerated KM tissue (Compared to the Control group, * indicates *p* < 0.05, ** indicates *p* < 0.01, and *** indicates *p* < 0.001; compared to the iPRF group, ^#^ indicates *p* < 0.05, ^##^ indicates *p* < 0.01, and ^###^ indicates *p* < 0.001; compared to the PGA group, ^@^ indicates *p* < 0.05, ^@@^ indicates *p* < 0.01, and ^@@@^ indicates *p* < 0.001).

To further evaluate the efficacy of the hydrogel in promoting KM regeneration, histological sections of the wound and surrounding tissue were analyzed via H&E and Masson staining. The PGAE group exhibited the most robust regeneration, characterized by abundant newly formed KM with a more continuous and organized epithelial layer (Figure 6E). Quantitative analysis revealed that the inter‐epithelial spacing in the PGAE group was significantly narrower than in other groups (Figure 7B). Moreover, the thickness of the neoepithelium in the PGAE group (89.63 ± 2.41 µm) substantially exceeded that in the Control (40.71 ± 5.31 µm), iPRF (47.00 ± 6.1 µm), and PGA (63.92 ± 8.65 µm) groups (Figure S18). Masson staining further demonstrated sparse, loosely arranged, and disorganized collagen fibers in both the Control and iPRF groups, whereas the PGAE group showed markedly enhanced collagen deposition with mature and well‐aligned fibrous structures (Figure 7A). Quantitative assessment confirmed that the collagen deposition thickness in the lamina propria of the PGAE group (634.1 ± 113.72 µm) was significantly greater than that in the Control (163.7 ± 12.3 µm), iPRF (345.9 ± 43.08 µm), and PGA (465.5 ± 13.72 µm) groups (Figure 7C). We further investigated the mechanism underlying PGAE hydrogel‐enhanced KM regeneration using immunofluorescence staining. The phenotype of oral mucosa is determined by the underlying connective tissue and its fibroblasts, with keratinization characteristics often adapting to changes in the lamina propria [42, 43, 44]. COL1 and FN are major ECM components in KM and play critical roles in regulating its phenotype. Unlike the elastic non‐keratinized oral mucosa, the lamina propria of KM contains abundant type I collagen fibers that anchor the epithelium to the underlying hard tissues, providing mechanical stability. Thus, COL1 and FN are important ECM components for supporting KM formation and maintaining the structural stability of the regenerated mucosa. Immunofluorescence staining of COL1 and FN revealed weak expression in the Control and iPRF groups, whereas the PGAE group exhibited the strongest signal, indicating that the hydrogel effectively upregulates COL1 and FN expression in the regenerated mucosa and promotes the formation of high‐quality keratinized tissue (Figure 7D–F). To evaluate the potential biosafety of the hydrogel in vivo, major organs, including the heart, liver, spleen, lungs, and kidneys, were collected on postoperative day 9 for H&E staining. Results showed no significant pathological changes in any of these organs across all groups, suggesting favorable preliminary in vivo biosafety of the PGAE hydrogel for KM repair (Figure S19).

In this study, a rat hard‐palatal mucosal defect model was used to evaluate the in vivo reparative effect of PGAE hydrogel. The hard‐palatal mucosa is a type of keratinized mucosa, characterized by keratinized stratified squamous epithelium and dense connective tissue, and shares certain histological and biological similarities with peri‐implant keratinized mucosa. Moreover, hard‐palatal mucosa is a commonly used and important autogenous soft tissue donor source for the treatment of insufficient peri‐implant keratinized mucosa in clinical practice. In addition, the hard palate is continuously exposed to the oral environment, including salivary flushing and mechanical stimulation, which allows preliminary evaluation of hydrogel integration and wound repair under a moist oral environment. Therefore, the hard‐palatal mucosal defect model can serve as a reproducible proxy model for preliminarily evaluating the feasibility of PGAE hydrogel in treating keratinized mucosal defects in the oral cavity and its potential to promote keratinized mucosa regeneration. However, this model does not fully reproduce the clinical peri‐implant soft tissue defect environment, particularly because it lacks a trans‐mucosal implant/abutment structure and cannot recapitulate the implant–soft tissue interface, peri‐implant biofilm challenge, or the complex mechanical loading patterns around dental implants. Therefore, the present in vivo results should be regarded as a proof‐of‐concept evaluation of keratinized mucosa regeneration. Further studies using peri‐implant defect models containing implant/abutment structures, especially in large animals, are needed to better assess the clinical applicability of PGAE hydrogel.

## 3 Conclusion

In summary, we have successfully developed a highly promising keratinized mucosa regeneration material—the PGAE hydrogel—based on iPRF‐MA, Alg‐NHS, and Lut@EGCG. At the material level, the hydrogel exhibits excellent fatigue resistance and robust wet tissue adhesion, enabling stable performance in the dynamic oral environment. Biologically, the PGAE hydrogel not only ameliorates the regenerative microenvironment of keratinized mucosa but also reprograms neutrophil function by blocking the ROS–MPO/NE axis, shifting the phenotype from NETosis to phagocytosis, thereby enabling specific clearance of oral biofilm infections. Moreover, the hydrogel enables dual modulation of gingival fibroblasts through both biochemical and biophysical signaling. It activates the FAK–RhoA–F‑actin–YAP pathway, promoting the expression of COL1 and FN and enhancing the synthesis of a keratinized mucosa‐like extracellular matrix. In vivo results demonstrate that the PGAE hydrogel significantly reduces bacterial accumulation in the regeneration site and upregulates COL1 and FN expression in the neomucosa, leading to high‐quality keratinized mucosa regeneration. This work establishes a versatile and effective strategy for treating keratinized mucosa deficiencies with considerable clinical potential.

## Conflicts of Interest

The authors declare no conflicts of interest.

## Supporting information




**Supporting File**: advs76188‐sup‐0001‐SuppMat.docx.

## Data Availability

The data that support the findings of this study are available from the corresponding author upon reasonable request
