# Supplementary material for: A Blood‐Derived Double‐Network Hydrogel with Robust Wet Adhesion for Keratinized Mucosa Regeneration via Neutrophil Phenotype Reprogramming and Mechanophysical Niche Modulation
Source: Adv Sci (Weinh). 2026 Jun 18:e76188. Online ahead of print. doi: 10.1002/advs.76188 (PMC13336970; doi:10.1002/advs.76188)
Supplement: Supplementary file 1 — Supporting File: advs76188‐sup‐0001‐SuppMat.docx. [file ADVS-9999-e76188-s001.docx]

**Supporting Information**

**A Blood-Derived Double-Network Hydrogel with Robust Wet Adhesion for Keratinized Mucosa Regeneration via Neutrophil Phenotype Reprogramming and Mechanophysical Niche Modulation**

*Sicong Ren^#^, Xinting Yang^#^, Jingxia Chen, Yihan Wang, Jian Feng, Jing Zhou, Jiaxin Luo, Jingjie Zhai*, Quan Lin*, Yanmin Zhou**

**1. Materials and methods**

**1.1. Materials**

The EGCG, Lut and Alg were purchased from Sigma (USA). Ultrapure water was prepared using a Milli-Q Plus system and used throughout the experiments. Gelatin methacryloyl (GelMA), 3-carboxyphenylboronic acid, and lithium phenyl-2,4,6-trimethylbenzoylphosphinate (LAP) were purchased from SUNP Biotech (Beijing, China). Dulbecco’s modified Eagle’s medium (DMEM), fetal bovine serum (FBS), Trypsin and penicillin/streptomycin were obtained from Hyclone (USA). Cell counting kit-8 (CCK-8) and Calcein acetoxymethyl ester/propidium iodide (Calcein-AM/PI) kit were obtained from Beyotime (Shanghai, China). 2′,7′-Dichlorofluorescin diacetate (DCFH-DA) and Human Neutrophil Isolation Kit were supplied by Solarbio (Beijing, China). CTL Plus luminescent cell viability assay kit and Mitochondrial membrane potential assay kit were obtained from Beyotime (Shanghai, China). Mito tracker green and Mito tracker red were obtained from Beyotime (Shanghai, China). TNFα, iNOS, Arg and IL10 Monoclonal antibody were purchased from Santa (USA). FAK, RHOA, YAP, COL1 and FN Monoclonal antibody were purchased from Beyotime (Shanghai, China). CD86 Polyclonal antibody and CD206 Monoclonal antibody were purchased from Proteintech (Wuhan, China). All other reagents were bought from local suppliers and utilized as received.

**1.2 Synthesis and characterization of PGAE hydrogel.**

**Synthesis of iPRF-MA**

For hydrogel characterization, vascularization-related in vitro assays, and experiments involving human gingival fibroblasts, hydrogels were prepared using human iPRF. After written informed consent was obtained, peripheral venous blood was collected from five healthy volunteers aged 20–30 years under aseptic conditions. Blood collection was approved by the Medical Ethics Committee of the Hospital of Stomatology, Jilin University (Approval No.: 2019-40). Blood samples were collected into 5 mL vacuum tubes without anticoagulant and centrifuged at 700 rpm for 3 min at room temperature. The yellow plasma fraction in the upper layer was collected as iPRF using a sterile syringe. For the remaining in vitro and in vivo experiments, rat-derived iPRF was used to prepare hydrogels. Rats were anesthetized with Avertin, and 5 mL of blood was collected by cardiac puncture into disposable vacuum blood collection tubes, followed by immediate centrifugation at 700 rpm for 3 min at room temperature. The yellow plasma fraction was collected as iPRF for subsequent use. Subsequently, 250 mg of freeze-dried GelMA and 250 μL of LAP solution were dissolved in 2.5 mL of PBS at 55 °C to obtain the GelMA precursor solution. Different proportions of iPRF were then added to the GelMA precursor solution to obtain iPRF-MA.

**Synthesis of Lut@EGCG microspheres**

EGCG (60 mg) and Lut (10 mg) were dissolved in 15 mL of 5% ethanol solution. The solution was adjusted to alkaline pH with NaOH, followed by the addition of 10 μL of anhydrous formaldehyde. After stirring for 15 min, 10 mg of glycine was added, and the mixture was stirred for another 4 h to allow the self-assembly of EGCG and Lut into Lut@EGCG microspheres. The microspheres were then collected by centrifugation at 10,000 rpm for 10 min and washed with deionized water.

**Synthesis of dual-network iPRF-MA/Alg-NHS/Lut@EGCG hydrogel**

Alg was first dissolved in deionized water at a concentration of 1 g in 15 mL. Equimolar amounts of EDC and NHS were then added to the Alg solution, and the pH was adjusted to 6.0. After reaction for 2 h, the solution was slowly added dropwise into 200 mL of ethanol to precipitate Alg-NHS by antisolvent precipitation. The precipitate was washed three times with deionized water and subsequently freeze-dried. To prepare the hydrogel precursor, 150 mg of Alg-NHS was dissolved in 5 mL of deionized water, and 2 mg of Lut@EGCG microspheres was dispersed in 1 mL of deionized water. Subsequently, 400 μL of the Alg-NHS solution and 200 μL of the Lut@EGCG dispersion were added to iPRF-MA precursor solutions containing different concentrations of GelMA. The mixture was stirred at room temperature for 2 h to ensure homogeneous mixing and then poured into molds to form hydrogels. The obtained hydrogels were freeze-dried and processed into blocks or powders for subsequent scanning electron microscopy and Fourier transform infrared spectroscopy analyses. In the following sections, iPRF-MA, iPRF-MA/Alg-NHS, and iPRF-MA/Alg-NHS/Lut@EGCG hydrogels are denoted as PG, PGA, and PGAE hydrogels, respectively.

**Characterization of Lut@EGCG**

EGCG or Lut@EGCG samples were dispersed in deionized water at a concentration of 1 mg in 10 mL by ultrasonication and then diluted to an appropriate concentration. The zeta potential was measured using a DTS1070 cell in Zeta mode, and the particle size distribution was measured using a DTS0012 cell in Size mode on a Zetasizer Nano ZS analyzer. EGCG and Lut@EGCG solutions were prepared at a concentration of 0.01 mg/mL and placed in quartz cuvettes for UV–vis spectroscopy using a Lambda 800 spectrophotometer. For SEM observation, lyophilized Lut@EGCG powder was redispersed in deionized water by ultrasonication for 10 min and dropped onto silicon wafers. After drying, the samples were sputter-coated with gold and observed using a JEOL JSM-6700F scanning electron microscope at 3 kV.

**Characterization of mechanical properties of hydrogel**

The rheological properties of the hydrogels were measured using a rheometer. A parallel-plate geometry with an 8 mm diameter was used, and the gap was set to 1000 μm. For strain sweep measurements, the temperature was maintained at 25 °C, the strain range was set from 0.1% to 1000%, and the angular frequency was fixed at 10 rad/s. For time sweep measurements, the strain was fixed at 1%, and the measurement was performed for 180 s. The mechanical properties of the hydrogels were evaluated using a universal testing machine equipped with a 100 N load cell. For compression tests, cylindrical hydrogel samples were compressed at a rate of 50 mm/min to a fixed strain of 60%. Shear adhesion tests were also performed using a universal testing machine equipped with a 100 N load cell.

**Characterization of wet tissue adhesion of hydrogel**

To evaluate wet tissue adhesion, a thin layer of water was applied to the surface of porcine skin using a pipette. Hydrogel precursor solutions with different compositions were then applied to the hydrated tissue surface and photocrosslinked under 405 nm light for 4 min. The samples were subjected to shear testing at a speed of 50 mm/min. Shear adhesion strength was calculated by dividing the maximum load by the contact area. In addition, hydrogel precursor solution was applied to wet porcine skin and photocrosslinked in situ to form a hydrogel. The in-situ gelation process and the resistance of the hydrogel to twisting and bending deformation were recorded photographically.

**Detection of cell proliferation promoting ability of hydrogel**

Three groups of sterile materials (iPRF, PGA and PGAE) were incubated in DMEM cell culture medium. After three days of incubation, supernatant was collected to prepare conditioned medium for subsequent experiments on fibroblasts, macrophages and HUVECs. Fibroblasts (L929 cells) were inoculated into 96-well plates, and the seeding density of each well was 5×10^3^ cells. After the cells attached, conditioned medium was added to each well. On the 1st, 2nd and 3rd day after culture, CCK-8 reagent was added and incubated in an incubator containing 5% CO_2_ for 2 hours. Then the absorbance of each well supernatant was detected at 450 nm by microplate reader. L929 cells were cultured as described above. After incubation for 3 days, the cells were stained with Calcein-AM/PI staining kit according to the kit instructions. Fluorescence images were obtained by confocal microscope.

**1.3 Detection of PGAE hydrogel's ability to regulate oral wound microenvironment**

**Detection of the vascularization ability of hydrogel**

**Cell scratch test:** human umbilical vein endothelial cells (HUVECs) were spread in a 6-well plate at a density of 1×10^6^/ well, and DMEM medium containing 10% serum was added, and it was cultured overnight in an incubator. Then it was changed to serum-free DMEM basal medium. After starvation for 12 h, a straight scratch was created using a sterile 200 μL pipette tip. The plates were washed three times with sterile PBS to remove suspended cells. Then the cells were divided into four groups: 1) Control group: serum-free DMEM medium was added; 2) iPRF group: adding serum-free iPRF conditioned medium; 3) PGA group: serum-free PGA conditioned medium was added; 4) PGAE group: serum-free PGAE conditioned medium was added. At 0, 24 and 48 h after scraping, the cells in the same field of view were photographed with inverted microscope, and the scratch area was calculated with ImageJ. Cell migration rate (%) = (A_0_-A_T_)/A_0_× 100%, where A_0_ is the initial scratch area at 0 h and A_T_ is the scratch area at the corresponding time point.

**Transwell experiment:** HUVECs were resuspended in DMEM medium containing 2% serum and added to the upper chamber of Transwell chamber with a density of 4×10^4^/ well. According to the different stimuli added to the lower chamber, it was divided into four groups: 1) Control group: DMEM medium containing 2% serum was added; 2) iPRF group: 300 μL iPRF and DMEM medium containing 2% serum were added; 3) PGA group: 300 μL PGA hydrogel and DMEM medium containing 2% serum were added; 4) PGAE group added 300 μL PGAE hydrogel and DMEM medium containing 2% serum. After being cultured in the incubator for 3 h, the cells were taken out, fixed with 4% paraformaldehyde for 10 min, washed with PBS for 3 times, dyed with crystal violet stain for 5 min, and then washed with PBS for 2 times. The cells in the upper chamber were gently wiped with a cotton swab, and the cells migrated to the bottom of the chamber were observed and recorded with an inverted microscope. Three views in each group were selected for counting.

**Tube-forming experiment:** put the Matrigel in a refrigerator at 4℃ for overnight melting, add the melted Matrigel into a 96-well plate, add 100 μL to each hole, and then put it in an incubator at 37℃ for 30 min to form gel. HUVECs were added into the prepared 96-well plate at a density of 2×10^4^ per well, and divided into 4 groups: Control group, iPRF group, PGA group and PGAE group. The Control group was added with DMEM complete medium, and the other groups were added with corresponding conditioned medium. After 6 h of culture, the cells on the gel were stained with Calcein-AM and observed under the fluorescence microscope. Finally, ImageJ is used to calculate various indexes of the tubular structure.

**Detection of anti-inflammatory ability of hydrogel**

**Free radical scavenging ability test:** 2,2-diphenyl-1-picrylhydrazyl (DPPH) and 2,2’-azino-bis (3-ethylbenzothiazoline-6-sulfonic acid) (ABTS) were tested. In the DPPH experiment, we first dissolved 4mg of DPPH reagent in 100 mL of ethanol, and then we put 10 mg of hydrogels with different components into 5mL of DPPH solution. After being kept in the dark at room temperature for a certain period of time, the wavelength of 517 nm was scanned by ultraviolet-visible absorption spectrometer. The DPPH clearance rate was calculated by the formula: DPPH clearance (%) = (C_0_-C_n_)/C_0_×100%, where C_0_ was the blank group and C_n_ was the experimental group. In ABTS experiment, 3 mL deionized water and 800 μL ABTS solution were added to 10 mg hydrogel, and then the ultraviolet absorption peak at 734 nm was measured after it was left in a dark environment at room temperature for a certain time. The clearance rate of ABTS was calculated by the formula: ABTS clearance (%) = (C_0_-C_n_)/C_0_× 100%, where C_0_ was the blank group and C_n_ was the experimental group.

**Reactive oxygen species (ROS) scavenging experiment:** ROS levels in cells were further detected using ROS fluorescence probes. Macrophages (RAW264.7) cells were divided into 5 groups, Blank group, Control group, iPRF group, PGA group and PGAE group. The macrophages were then seeded on a 24-well plate at a density of 1×10^5^ per well. After the cells were attached, the Control group, iPRF group, PGA group and PGAE group added DMEM complete medium containing lipopolysaccharide (LPS concentration was 1 μg/mL). After 3 h of incubation, the Control group was replaced with DMEM medium, and the iPRF group, PGA group and PGAE group were replaced with corresponding conditioned medium. Complete DMEM culture medium was used throughout the Blank group. After 24 hours of culture, the cell culture medium was removed and a 2,7-dichlorofluorescein diacetate (DCFH-DA) probe was added according to the instructions. After incubation in the incubator for 30 minutes, the culture medium was removed, the PBS was rinsed 3 times, and then stained with Hoechst 33342 staining solution for 5 minutes, and finally the ROS content of the cells was checked using fluorescence microscopy.

**Inflammation-related gene expression detection:** Real-time quantitative polymerase chain reaction (RT-qPCR) was used to evaluate the effect of different groups of hydrogels on the expression of inflammation-related genes in macrophages. The macrophages were then seeded on a 24-well plate at a density of 1×10^5^ per well. After the cells were attached, the Control group, iPRF group, PGA group and PGAE group added DMEM complete medium containing LPS (LPS concentration was 1μg/mL). After 3 h of incubation, the Control group was replaced with DMEM medium, and the iPRF group, PGA group and PGAE group were replaced with corresponding conditioned medium. Complete DMEM culture medium was used throughout the Blank group. After the culture was completed, the RNA of the cells was extracted by Trizol and reverse transcription was performed by the TAKARA reverse transcription kit as directed. The mass and concentration of RNA were calculated by Thermo nano drop 2000 c. Finally, polymerase chain reaction was performed by the RT-qPCR kit and the Applied Biosystems 7300 as per instructions. Gene primers are shown in **Table S1**. Data normalized to expression of glyceraldehyde-3-phosphate dehydrogenase (GAPDH) were analyzed by the 2-ΔΔCq method, and each experiment was repeated 3 times.

**Immunofluorescence staining:** Immunofluorescence was used to detect the expression of inducible nitric oxide synthase (‌iNOS), TNFα, IL10 and arginine (Arg) at the protein level to evaluate the effect of iPRF-MA/Alg-NHS/Lut@EGCG on macrophage polarization. RAW264.7 cells were divided into 5 different groups, Blank group, Control group, iPRF group, PGA group and PGAE group. The macrophages were then seeded on a 24-well plate at a density of 10×10^4^ per well. After the cells were attached, the Control group, the iPRF group, the PGA group and the PGAE group added the DMEM complete medium containing LPS (the LPS concentration was 1 μg/mL). After 3 h of incubation, the Control group was replaced with DMEM medium, and the iPRF group, the PGA group and the PGAE group were replaced with the corresponding conditioned medium. Complete DMEM culture medium was used throughout the Blank group. After removing the cell culture medium, the cells were fixed with 4% paraformaldehyde for 30min, washed 3 times with PBS, and incubated in immunostaining blocking buffer for 2 h. The cells were then incubated overnight at 4℃ using TNFα primary antibody, IL10 primary antibody, iNOS primary antibody and Arg primary antibody. IgG secondary antibody coupled with Alexa 488 and Alexa 647 was subsequently incubated at room temperature for 1 h. Nuclei were stained with DAPI for 6 min. Finally, a fluorescence image was obtained by a confocal microscope.

**Flow cytometry:** RAW264.7 cells were seeded on 6-well plates at a density of 5×10^5^ per well, and the cell grouping and culture method were the same as above. After 24 h of culture, each group of cells was collected in a centrifuge tube with a cell scraper and co-incubated with CD206 antibody on ice for 1 h. The results were then analyzed using FlowJo software.

**1.4 Phenotype regulation effect of PGAE hydrogel on neutrophils**

**Extraction of neutrophils**

Neutrophils from human peripheral blood were extracted using a neutrophil isolation kit. Blood collection was approved by the Medical Ethics Committee of the School of Stomatology, Jilin University (2019-40). First, 4 mL of intravenous blood was collected into the heparin blood collection vessel, 4 mL of reagent A was added first, and then 2 mL of reagent C was slowly added to reagent A to form a gradient interface. The centrifuge tube was centrifuged at a speed of 1000 g for 30 minutes, the neutrophil layer was absorbed, the cell washing solution was added to wash the cells, and then centrifuged at a speed of 250 g for 10 minutes, and the neutrophils were collected for subsequent experiments.

**Preparation of RFH medium and conditioned medium**

FeSO_4_ (40μM) and H_2_O_2_ (400μM) were added to antibiotic-free RPMI-1640 complete medium to prepare "RFH medium" that simulates a high oxidative stress environment. RFH medium was then used in neutrophil-related experiments. Three groups of sterile materials (iPRF, PGA and PGAE) were incubated in antibiotic-free RPMI-1640 cell culture medium, and supernatant was collected after 3 days to prepare conditioned medium for subsequent neutrophil-related experiments.

**Neutrophil membrane staining**

Neutrophils were seeded at a density of 1×10^6^ per well into a 24-well plate containing poly-L-lysine-coated glass coverslips and divided into the following 5 groups, Blank group, Control group, iPRF group, PGA group and PGAE group. The Blank group used RPMI-1640 complete culture medium without antibiotics throughout the experiment. For the Control group, RFH medium was added to each well, and after incubation for 3 h, it was replaced with RPMI-1640 complete medium without antibiotics. For the iPRF group, PGA group and PGAE group, RFH culture medium and corresponding conditioned medium were added to each well, and then replaced with the corresponding conditioned medium after incubation for 3 h. After 3 h of culture, the cell membrane was stained with DiI probe and the nucleus was stained with Hoechst 33342. After the staining is completed, the coverslips loaded with cells are removed and the staining results are observed using a laser confocal microscope.

**Neutrophil NETs generation experiment**

Neutrophils were seeded at a density of 1×10^6^ per well into a 24-well plate containing poly-L-lysine-coated glass coverslip and divided into the following 5 groups, Blank group, Control group, iPRF group, PGA group and PGAE group. The Blank group used RPMI-1640 complete culture medium without antibiotics throughout the experiment. For the Control group, RFH medium was added to each well, and after incubation for 3 h, it was replaced with RPMI-1640 complete medium without antibiotics. For the iPRF group, PGA group and PGAE group, RFH culture medium and corresponding conditioned medium were added to each well, and then replaced with the corresponding conditioned medium after incubation for 3 h. After the culture was completed, Annexin V-mCherry and SYTOX Green reagents were mixed in a ratio of 5:1, and then incubated with each group of cells for 30 min protected from light to stain the phosphatidylserine and NETs of the neutrophils. The nucleus was stained with Hoechst 33342. After the staining was completed, the coverslips loaded with cells were taken out and the staining results were observed using a laser confocal microscope.

**Quantitative analysis of NETs**

Neutrophils were seeded at a density of 1×10^6^ per well into a 24-well plate containing poly-L-lysine-coated glass coverslip and divided into the following 5 groups, Blank group, Control group, iPRF group, PGA group and PGAE group. The Blank group used RPMI-1640 complete culture medium without antibiotics throughout the experiment. For the Control group, RFH medium was added to each well, and after incubation for 3 h, it was replaced with RPMI-1640 complete medium without antibiotics. For the iPRF group, PGA group and PGAE group, RFH culture medium and corresponding conditioned medium were added to each well, and then replaced with the corresponding conditioned medium after incubation for 3 h. After the culture was completed, neutrophils and culture medium were carefully absorbed into the EP tube and centrifuged at 4℃ to collect the supernatant containing NETs, stain the supernatant containing NETs using Hoechst 33342, and absorbance was determined at 465nm using a microplate reader.

**Neutrophil reactive oxygen generation assay**

ROS levels in neutrophils were detected using ROS fluorescence probe (DCFH-DA). Neutrophils were seeded at a density of 1×10^6^ per well into a 24-well plate containing poly-L-lysine-coated glass coverslip and divided into the following 5 groups, Blank group, Control group, iPRF group, PGA group and PGAE group. The Blank group used RPMI-1640 complete culture medium without antibiotics throughout the experiment. For the Control group, RFH medium was added to each well, and after incubation for 3 h, it was replaced with RPMI-1640 complete medium without antibiotics. For the iPRF group, PGA group and PGAE group, RFH culture medium and corresponding conditioned medium were added to each well, and then replaced with the corresponding conditioned medium after incubation for 3 h. After the culture is completed, the DCFH-DA probe is added according to the instructions. After incubation in the incubator for 30 minutes, the culture medium was removed, and then stained with Hoechst 33342 staining solution for 5 minutes. After the staining was completed, the coverslips loaded with cells were removed, and finally the ROS content of the cells was checked using a fluorescence microscope.

**Immunofluorescence staining**

The expression of MPO and NE in neutrophils was detected by immunofluorescence. Neutrophils were seeded into a 24-well plate containing poly-L-lysine-coated glass coverslip at a density of 1×10^6^ per well, and divided into the following 5 groups, Blank group, Control group, iPRF group, PGA group and PGAE group. The Blank group used RPMI-1640 complete culture medium without antibiotics throughout the experiment. For the Control group, RFH medium was added to each well, and after incubation for 3 h, it was replaced with RPMI-1640 complete medium without antibiotics. For the iPRF group, PGA group and PGAE group, RFH culture medium and corresponding conditioned medium were added to each well, and then replaced with the corresponding conditioned medium after incubation for 3 h. After the culture was completed, the cell culture medium was removed, the cells were fixed with 4% paraformaldehyde for 30 minutes, slowly rinsed with PBS 3 times, and incubated in immunostaining blocking buffer for 2 h. The cells were then incubated overnight with MPO primary antibody and NE primary antibody. Subsequently, IgG secondary antibody coupled with Alexa 488 was incubated at room temperature for 1 h. Then, the configured phalloidin working solution and 500 μL of DAPI staining solution were added to each well to stain the cytoskeleton and nucleus. Finally, a fluorescence image was obtained by a confocal microscope.

**1.5 Effect of PGAE hydrogel on antibacterial ability of neutrophils**

**Bacterial culture**

Porphyromonas gingivalis (*P. gingivalis*) and Streptococcus mutans (*S. mutans*) were cultured using BHI medium (BHI powder 36 g L^-1,^ yeast powder 5 g L^-1^, L-cysteine 0.5g L^-1^, vitamin K 1 mg L^-1^, heme chloride 5 mg L^-1^). The bacteria were cultured under anaerobic conditions at 37℃. After the bacteria grow to the logarithmic growth period, adjust the concentration of the bacterial solution to 1×10^8^ CFU mL^-1^ for subsequent experiments. For the formation of single-species biofilms of *P. gingivalis* and *S. mutans*, the concentration of the logarithmic bacterial fluid was adjusted to 1×10^8^ CFU mL^-1^, and the circular coverslips were placed at the bottom of a 24-well plate and cultured for 72 h in anaerobic conditions to form the biofilm. For the formation of biofilms of multi-bacterial species, the concentration of the mixed bacterial solution was adjusted to 1×10^8^ CFU mL^-1^ after mixing in equal proportions, and the biofilm was cultured in anaerobic conditions for 96 h.

**Experiment grouping**

(1) Blank group: Bacteria were treated with untreated neutrophils, denoted as "Blank"; (2) Control group: Bacteria were treated with neutrophils cultured with RFH medium, denoted as "Control";

(3) iPRF group: Bacteria were treated with neutrophils cultured with RFH medium and iPRF conditioned medium;

(4) PGA group: Bacteria were treated with neutrophils cultured with RFH medium and PGA conditioned medium;

(5) PGAE group: Bacteria were treated with neutrophils cultured with RFH medium and PGAE conditioned medium;

**Biofilm disruption assay**

After the treatment was completed, the coverslips were collected, and after washing PBS, they were fixed with 2% glutaraldehyde, and the biofilm was stained with crystal violet solution. After 20 minutes, the dye solution was removed, washed with PBS and dried and scanned for photos.

**Colony-forming units (CFU) count**

The above-mentioned cultured biofilms were transferred to centrifuge tubes and bacteria were obtained on the biofilm by pipetting and vortexing. Take 10 μL of bacterial suspension and dilute it by gradient dilution and inoculate it on blood agar plates. The samples were cultured under anaerobic conditions at 37 °C for an appropriate period. The number of colonies was calculated based on the colony counts on blood agar plates and the corresponding dilution factor.

**Fluorescent staining of bacterial biofilm**

Neutrophils were first stained with CellTracker Blue. Then, labeled neutrophils (5×10^6^ cells/well) were carefully added to the prepared biofilm. After 3 h, gently remove the culture medium, and the mixture of SYTO9 (2.5 μM) and propidium iodide (2.5 μM) prepared in advance were dropped on the surface of the biofilm and incubated for 15 min away from light. 3D images of biofilm were observed using laser confocal microscope.

**1.6 Bio-Physiochemical dual regulation of PGAE hydrogel on Human gingival fibroblasts (HGFs)**

**Isolation and culture of HGFs**

Volunteers aged 18–25 years were recruited from the Oral Implant Center of the Hospital of Stomatology, Jilin University. After written informed consent was obtained, gingival tissue samples were collected under sterile conditions using a gingival knife. The study was conducted in accordance with the Declaration of Helsinki and was approved by the Medical Ethics Committee of the Hospital of Stomatology, Jilin University (Approval No.: JDKQ202372). The gingival tissues were repeatedly washed with DMEM supplemented with penicillin–streptomycin and thoroughly digested for 2 h. The epithelial layer was then removed, and the connective tissue was retained for subsequent experiments. The tissue was cut into approximately 1 mm³ fragments and placed at the bottom of culture flasks. After tissue attachment, DMEM complete medium was gently added. When cells migrated out from the tissue explants and reached approximately 80% confluence, they were passaged using 0.25% trypsin. Cells at passages 3–6 were used for subsequent experiments.

**Cell colony formation experiment**

HGFs were seeded into a 6-well plate at a low density of 2×10^3^ per well, and after the cells were attached, they were divided into the following 4 groups, the Control group, the iPRF group, the PGA group and the PGAE group. The Control group was cultured using DMEM complete medium throughout the experiment, while the iPRF group, PGA group and PGAE group were cultured using the corresponding conditioned medium. After 7 days of culture, the cells in the wells were stained with crystal violet solution. After 10 minutes, the crystal violet staining solution was removed and washed three times with PBS. Then the images were recorded using a scanner and the colony area was quantitatively analyzed.

**Scratch experiment**

HGFs were seeded in a 6-well plate at a density of 1×10^5^ per well, and the cells reached approximately 90% confluence. Replace the medium with serum-free DMEM basal medium. After 12 h of starvation treatment, a straight scratch was created using a sterile 200 μL pipette tip. Wash the plates 3 times with sterile PBS to remove suspended cells. Then the cells were divided into 4 groups: 1) Control group: Add serum-free DMEM medium; 2) iPRF group: Add serum-free iPRF conditioned medium; 3) PGA group: Add serum-free PGA conditioned medium; 4) PGAE group: Add serum-free PGAE conditioned medium. The cells were then stained with Calcein-AM, and the same positions were taken at 0 and 12 h, respectively, using a laser confocal microscope, and the scratch area was calculated using ImageJ. Cell migration rate (%) = (A_0_-A_T_)/A_0_×100%, where A_0_ is the initial scratch area of 0 h and A_T_ is the scratch area of 12 h.

**Observe the growth of HGFs within hydrogels**

The hydrogel in the liquid phase was injected into the well plate, and 1mL was injected per well. The iPRF hydrogel formed spontaneously through coagulation, whereas the PGA and PGAE hydrogels were formed by photocrosslinking. After the curing was complete, the HGFs were seeded on the hydrogel at a density of 1×10^4^ per well. After 3 days of culture, the hydrogel in the well plate was taken out and stained with Calcein-AM for 30 minutes. After the staining was completed, the cells in the hydrogel were washed 3 times with PBS. Finally, the cells in the hydrogel were observed using laser confocal microscope.

**RNA sequencing**

After cell culture was completed, total RNA of HGFs in hydrogel was extracted using RNAiso reagent for RNA isolation and library preparation. RNA purity and quantification were evaluated using a NanoDrop2000 spectrophotometer and RNA integrity was evaluated using an Agilent2100 bioanalyzer. The transcriptome library was constructed using the VAHTS Universal V5 RNA-seq Library Prep Kit according to the manufacturer's instructions. The library was sequenced using the llumina Novaseq 6000 sequencing platform, resulting in a paired end read of 150 bp. The original readings in fastq format were processed using Fastp software and clean readings were obtained after removing low-quality readings for subsequent data analysis. Use HISAT2 to map clean reads to the reference genome. The number of bases (FPKM) of the exon model per million fragment per gene was calculated, where FPKM<1 was defined as low expression, 1≤FPKM<10 was defined as medium expression, and FPKM ≥10 was defined as high expression. Reads for each gene were obtained by HTSeq-count. Principal component analysis (PCA) was performed using R (v3.2.0) to evaluate the biological repetition of the samples. Differentially expressed genes were analyzed using DESeq2 software. Genes satisfying the thresholds of p < 0.05 and fold change > 2 or < 0.5 were defined as differentially expressed genes (DEGs). Systematic clustering analysis of DEGs was performed using R (v3.2.0) to demonstrate the expression patterns of genes in different groups and samples. Based on the hypergeometric distribution algorithm, Gene ontology (GO) and Kyoto encyclopedia of genes and genomes (KEGG) pathway enrichment analysis was performed on DEGs, respectively, to screen significant enrichment terms using R (v3.2.0).

**Expression of genes related to HGFs mechanical signaling pathway in hydrogels**

Different types of hydrogels were injected into 6-well plates, and 3 mL was injected per well. After complete curing, HGFs were inoculated in well plates at a density of 2×10^5^ per well. After 3 days of culture, the hydrogel in the well plate was removed, and the total RNA of HGFs in the hydrogel was extracted using RNAiso reagent, and reverse transcription was performed using the TAKARA reverse transcription kit as instructed. The mass and concentration of RNA were calculated by Thermo nano drop 2000 c. Finally, polymerase chain reaction was performed by the RT-qPCR kit and the Applied Biosystems 7300 as per instructions. Data normalized to GAPDH expression were analyzed by the 2^-ΔΔ^ Cq method. Each experiment was repeated three times. The primer list is shown in Table S2.

**Immunofluorescent staining of mechanical signaling pathway-associated proteins**

Different types of hydrogels were injected into 24-well plates, and 1mL was injected per well. After complete curing, HGFs were inoculated on the hydrogels in the well plate at a density of 1×10^4^ per well. After 3 days of culture, the hydrogel in the well plate was removed and the cells in the hydrogel were fixed for 30 minutes using paraformaldehyde. After fixation is completed, clean PBS 3 times for 3 minutes each time. The cells in the hydrogel were then blocked for 1 h using an immunostaining blocking solution. After the blocking is completed, the blocking solution is aspirated and the primary antibody diluted at 1:200 is added, as well as the configured phalloidin working solution. Incubate overnight in a 4℃ refrigerator and wash 3 times with PBS for 10min each time after incubation. Then add the diluted fluorescently labeled secondary antibody and incubate for 2 hours at room temperature away from light. After the incubation is completed, wash it with PBS for 10 minutes each time. Then add 500 μL of DAPI staining solution per well to stain the cell nucleus. After the staining was completed, the hydrogel was taken out from the well plate, and the protein expression of cells in the hydrogel was observed using a laser confocal microscope. Then, various indicators were analyzed using ImageJ software.

**1.7 In vivo experiments**

**Establishment of a keratinized mucosal defect model in rats**

36 male Sprague-Dawley rats weighing about 250 g were selected to construct a keratinized mucosal defect model to evaluate the effect of hydrogels to promote keratinized mucosal regeneration. All animal experiments were performed in accordance with the ethical guidelines for laboratory animal welfare and were approved by the Animal Care and Use Committee (CKARI202315). After two weeks of stable feeding, all rats were randomly divided into 4 groups: Control group, iPRF group, PGA group and PGAE group. The rats were fasted for 12 h before surgery. To establish an animal model of keratinized mucosal defect, rats were weighed and anesthetized with 1% pentobarbital. The oral cavity was opened with a mouth gag to expose the hard palate. A circular wound was created on the hard palate of each rat using a 4 mm biopsy punch, and central tissue was removed using a periosteal separator. Finally, hydrogel was injected at the defect site to cover the wound, the PGA and PGAE hydrogels were photocrosslinked in situ, the iPRF hydrogel was allowed to gel naturally, and the Control group was given normal saline at the wound. After recovery from anesthesia, the rats were returned to individual cages and provided with soft food for 3 days. After the experiment was completed, all rats were euthanized with an overdose of sodium pentobarbital. The wound and surrounding mucosal tissue were collected for subsequent Hematoxylin-Eosin (HE) staining and Masson staining. In addition, immunofluorescent staining (COL1 and FN) was performed to evaluate the effect of keratinized mucosal regeneration. To assess the potential biotoxicity of the hydrogel, we collected major organs including the heart, liver, spleen, lung and kidney and used HE staining for histological analysis.

**General observation of keratinized mucosal defect area and wound healing rate analysis.**

Wound images were taken on days 0, 3, 5, 7 and 9 after surgery, respectively, to observe tissue healing. The obtained images were analyzed using ImageJ software. Calculate wound healing rates for different groups according to the formula. Calculation formula: wound healing rate = (original area-unhealed area)/original area × 100%.

**Evaluation of antibacterial performance in hydrogels**

To evaluate the in vivo antibacterial effect, bacterial samples were collected from the wound sites using sterile swabs. The swabs were immediately placed into sterile EP tubes, and the collected samples were inoculated into liquid culture medium and incubated at 37 °C for 48 h. The bacterial suspensions were then serially diluted, and 100 μL of each dilution was evenly spread onto blood agar plates. After incubation, colonies were counted to determine bacterial load.

**HE staining**

Paraffin-embedded tissue sections were deparaffinized in xylene and rehydrated through a graded ethanol series. The sections were stained with hematoxylin for 5 min, rinsed briefly under running tap water for 5 s, and differentiated in 0.6% acid alcohol for 5 s. The sections were then immediately blued in 0.6% ammonia solution for 10 s. After rinsing under running tap water for 3 min, the sections were counterstained with eosin for 2 min. Subsequently, the sections were dehydrated through a graded ethanol series, cleared in xylene, and mounted with neutral resin mounting medium. Histological evaluation was performed under a light microscope, and quantitative histomorphometric analysis was conducted using ImageJ software.

**Masson staining**

Paraffin-embedded tissue sections were deparaffinized in xylene and rehydrated through a descending ethanol series. The sections were then stained according to the standard Masson’s trichrome staining protocol. Briefly, nuclei were stained with hematoxylin for 5 min, followed by rinsing under running tap water for 5 min. The sections were differentiated in 1% acid alcohol for 5 s and rinsed under running tap water for 30 s to stop differentiation. After bluing in 0.5% ammonia solution for 3 min, the sections were rinsed under running tap water for 10 min. Cytoplasmic staining was performed with Ponceau S–acid fuchsin solution for 5–8 min, followed by a brief rinse with ultrapure water. Collagen differentiation was performed using 1% phosphomolybdic acid solution for 1–3 min, followed by rinsing with ultrapure water. Collagen fibers were then stained with 2.5% aniline blue solution for 5 min, and the reaction was stopped by rinsing with ultrapure water. After staining, the sections were dehydrated through an ascending ethanol series, cleared in xylene, and mounted with neutral resin mounting medium. Histological evaluation was performed under a light microscope, and the collagen fiber area fraction was quantitatively analyzed using ImageJ software.

**Immunofluorescence staining**

Sections were deparaffinized in xylene, rehydrated through a graded ethanol series, and washed three times with TBST buffer for 5 min each. Antigen retrieval was performed using pepsin digestion solution at 37 °C for 30 min, followed by three washes with TBST. The sections were incubated with endogenous peroxidase blocker for 10 min at room temperature and washed again with TBST. Primary antibodies against COL1 and FN, diluted at 1:200, were added and incubated overnight at 4 °C in a humidified chamber. After washing with TBST, the sections were incubated with secondary antibodies diluted at 1:500 for 1 h at room temperature. Nuclei were counterstained with DAPI for 5 min, and the sections were mounted with antifade mounting medium. Fluorescence images were acquired using an inverted fluorescence microscope, and semi-quantitative analysis was performed using ImageJ software.

**1.8 Statistical analysis**

Statistical analysis was performed using a two-tailed Student’s t-test for comparisons between two groups or one-way analysis of variance (ANOVA) for comparisons among multiple groups. Data are presented as the mean ± standard deviation. Differences were considered statistically significant at *p* < 0.05.

**2. Results**

**Figure S1.** Zeta potential results of Lut@EGCG microspheres.


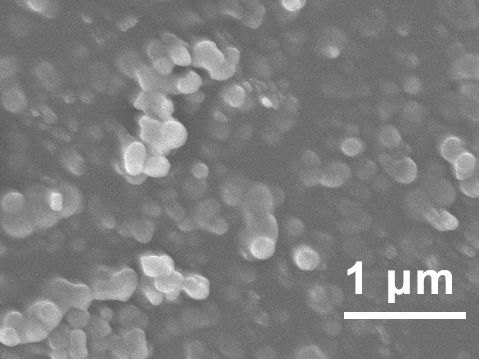


**Figure S2**. SEM images of Lut@EGCG microspheres.

**Figure S3.** Particle size analysis of Lut@EGCG microspheres.


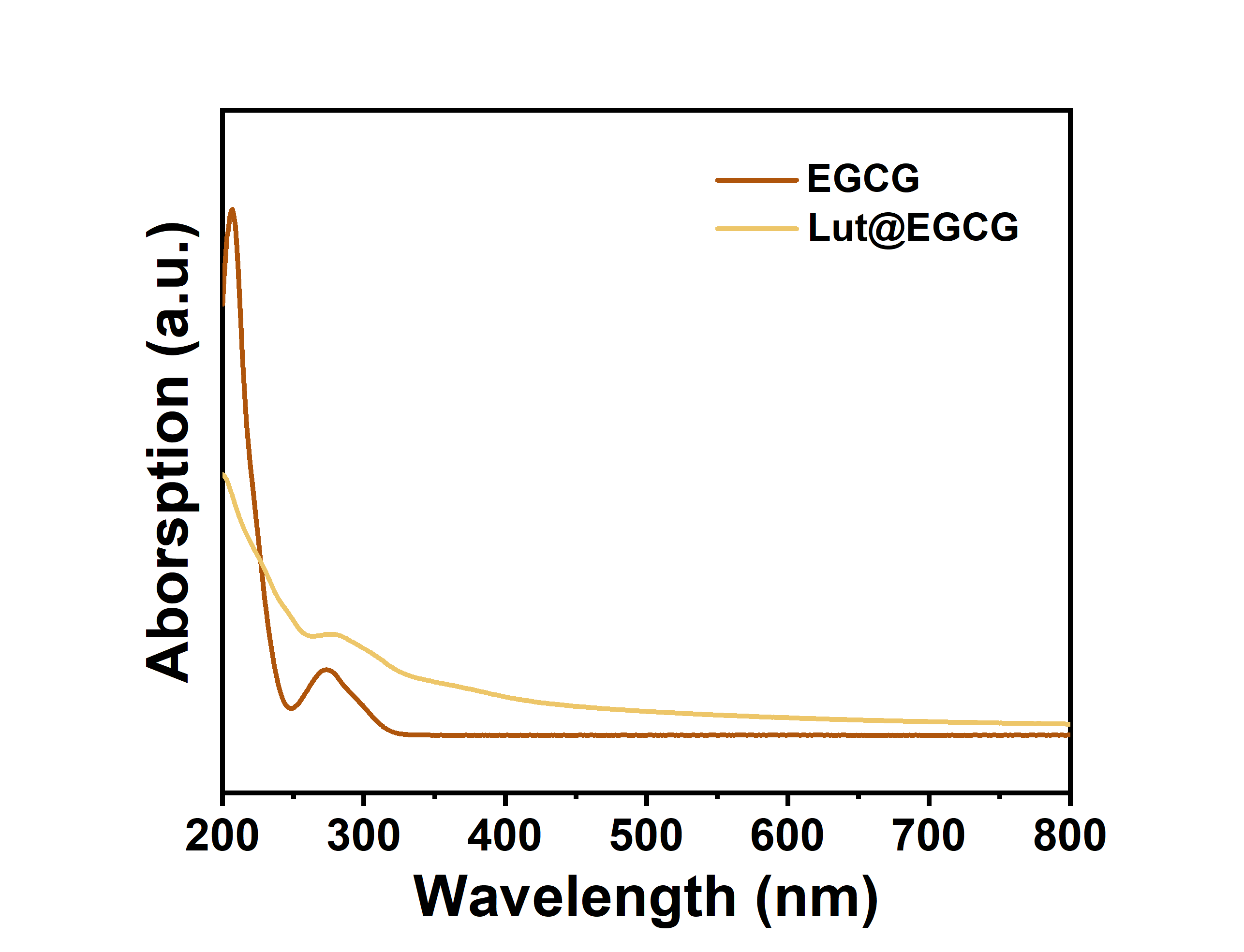


**Figure S4.** Ultraviolet-visible spectra of EGCG and Lut@EGCG microspheres.

**Figure S5.** Rheology test results of PGAE hydrogel.

**Figure S6.** The swelling ratio of PGAE hydrogels.

**Figure S7.** The modulus of PGAE hydrogels changes with 405 nm light irradiation time.


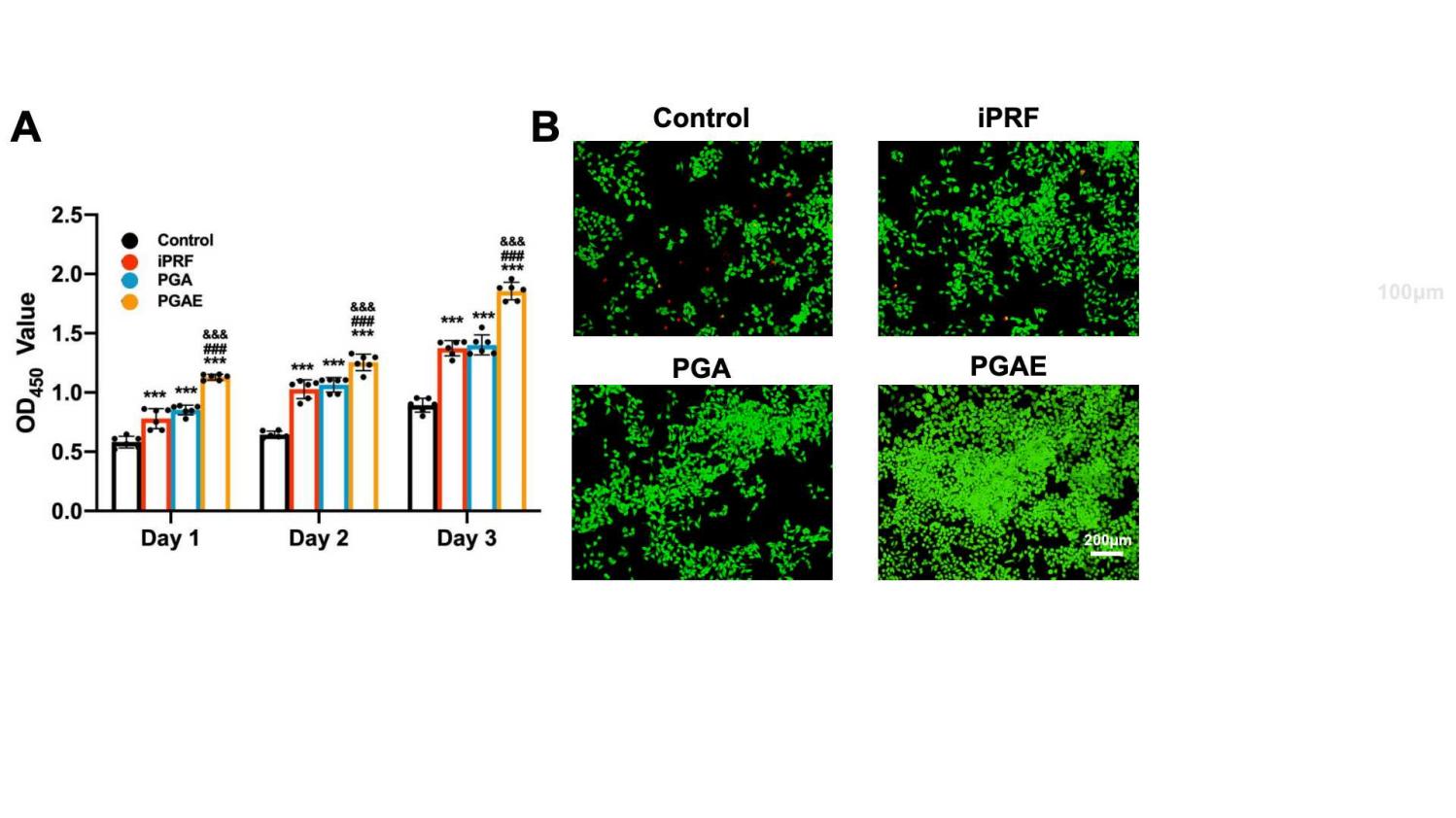


**Figure S8.** Effects of different hydrogels on cell proliferation. (A) CCK-8 results of different groups of cells at days 1, 2 and 3; (B) Live and dead staining results of different groups of cells at day 3. (Compared with the Control group, "*" means *p* < 0.05, "**" means *p* < 0.01, and "***" means *p* < 0.001; Compared with iPRF group, "#" means *p* < 0.05, "##" means *p* < 0.01 and "###" means *p* < 0.001; Compared with PGA group, "&"means *p* < 0.05, "&&"means *p* < 0.01 and "&&&"means *p* < 0.001.)


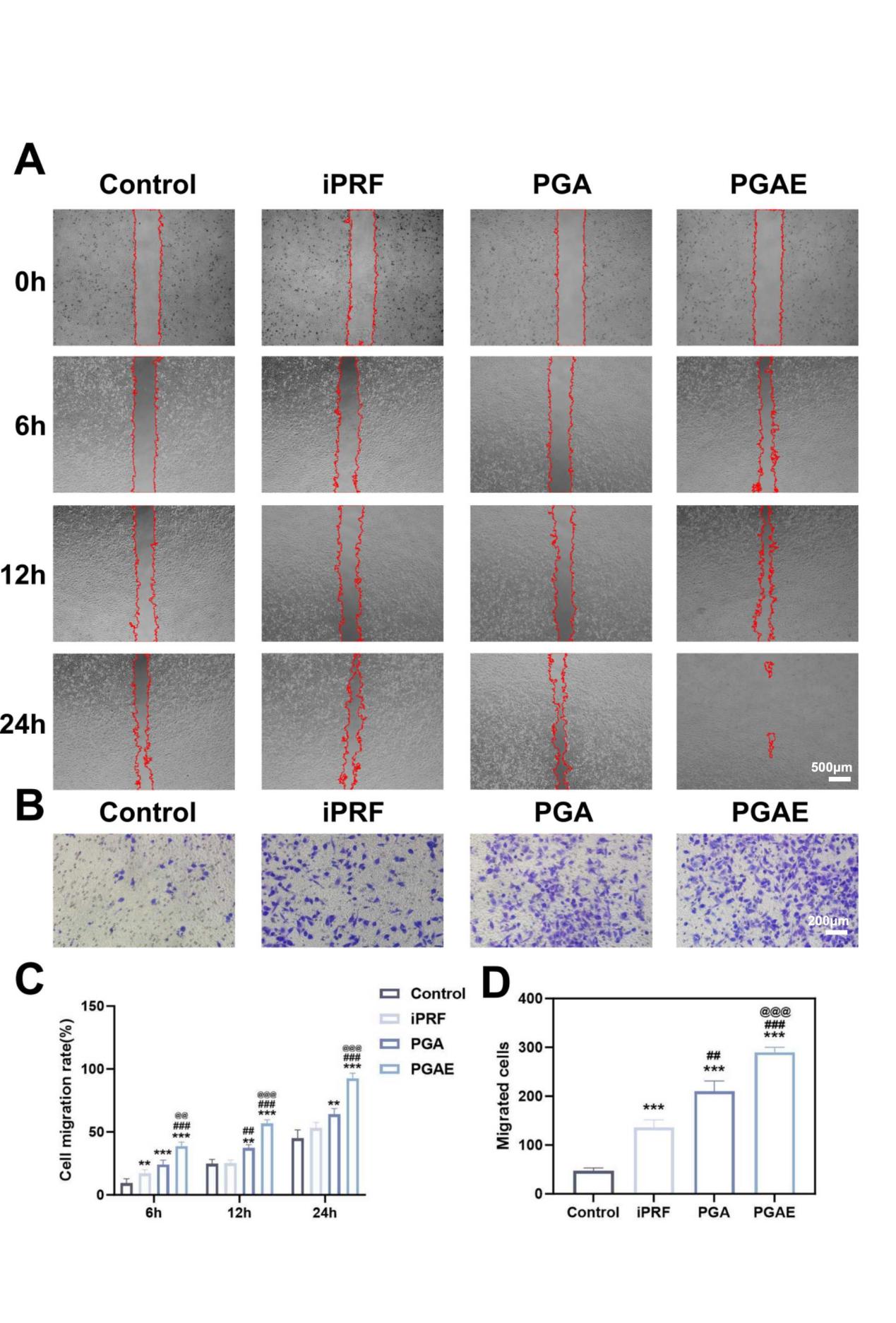


**Figure S9.** Effects of different hydrogels on HUVECs migration ability. (A) Cell scratch experiment results; (B) Transwell experiment results; (C) Cell migration rate in the scratch assay; (D) Number of HUVECs recruited by different hydrogels. (Compared with the Control group, "*" means *p* < 0.05, "**" means p < 0.01, and "***" means *p* < 0.001; Compared with iPRF group, "#" means *p* < 0.05, "##" means *p* < 0.01 and "###" means *p* < 0.001; Compared with PGA group, "@"means *p* < 0.05, "@@"means *p* < 0.01 and "@@@"means *p* < 0.001.)


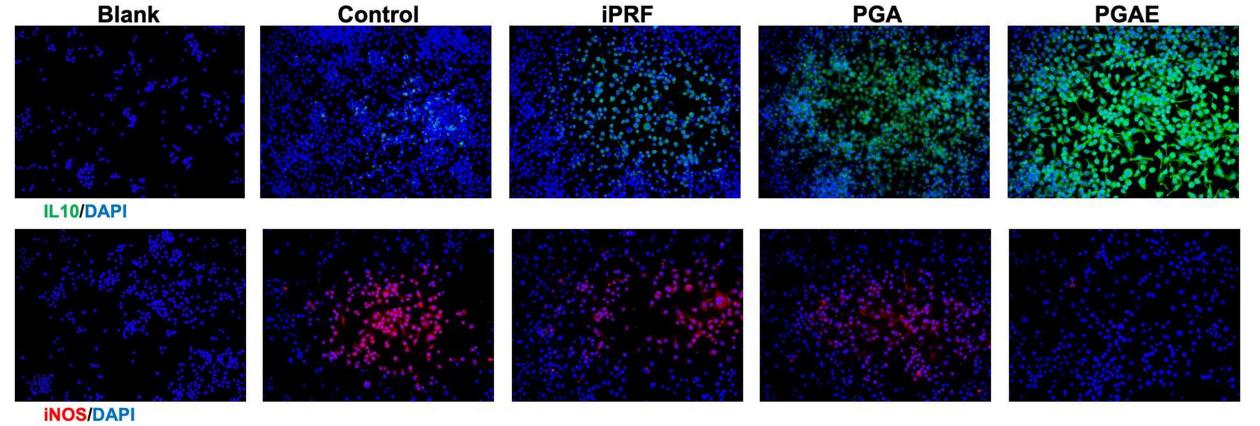


**Figure S10.** Fluorescence staining results of macrophage inflammation-related proteins.


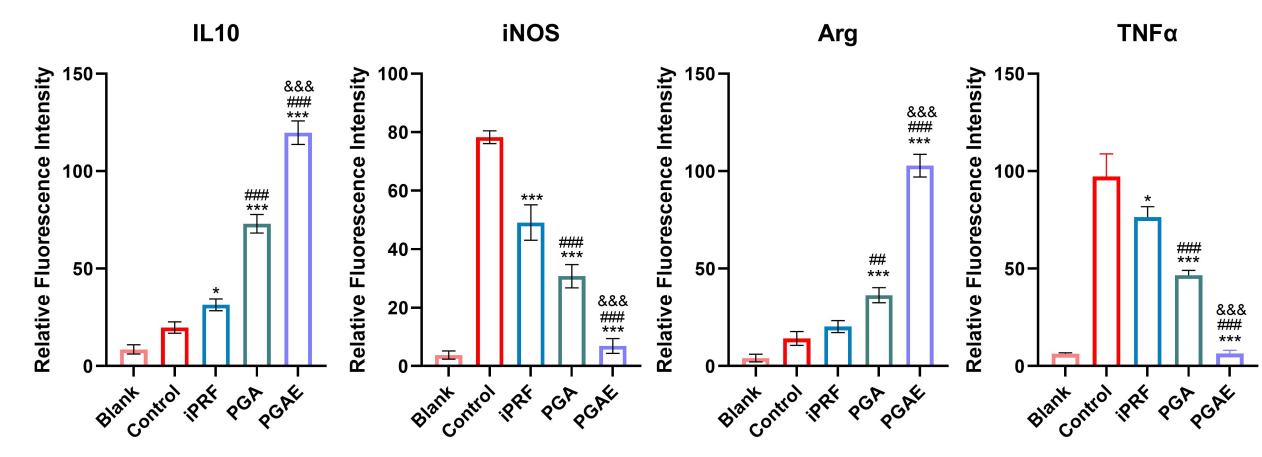


**Figure S11.** Quantitative analysis of fluorescence intensity. (Compared with the Control group, "*" means *p* < 0.05, "**" means *p* < 0.01, and "***" means *p* < 0.001; Compared with iPRF group, "#" means *p* < 0.05, "##" means *p* < 0.01 and "###" means *p* < 0.001; Compared with PGA group, "&"means *p* < 0.05, "&&"means *p* < 0.01 and "&&&"means *p* < 0.001.)


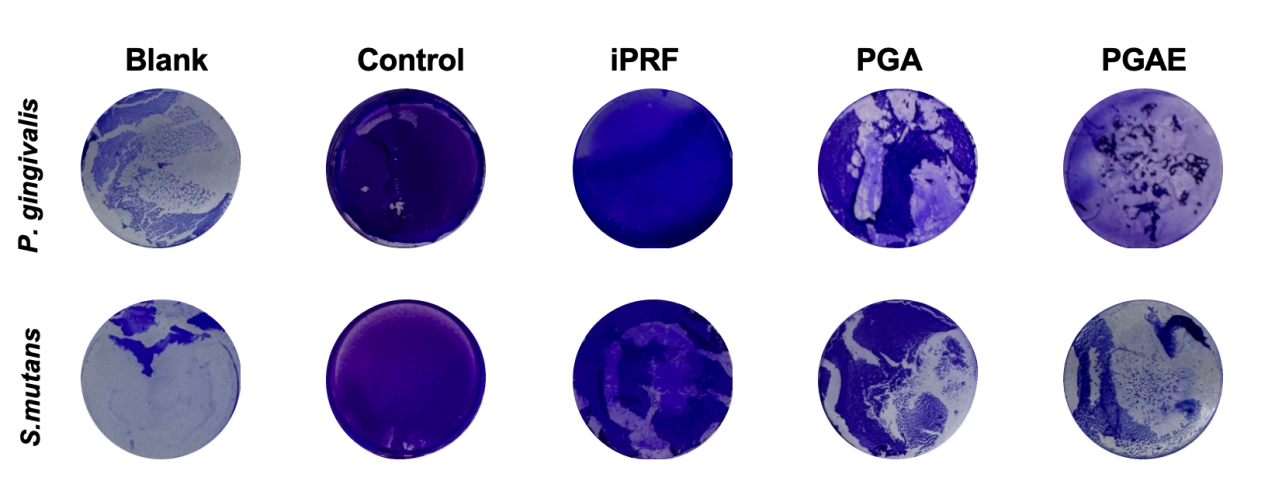


**Figure S12.** Effects of hydrogel-treated neutrophils on bacterial biofilms.


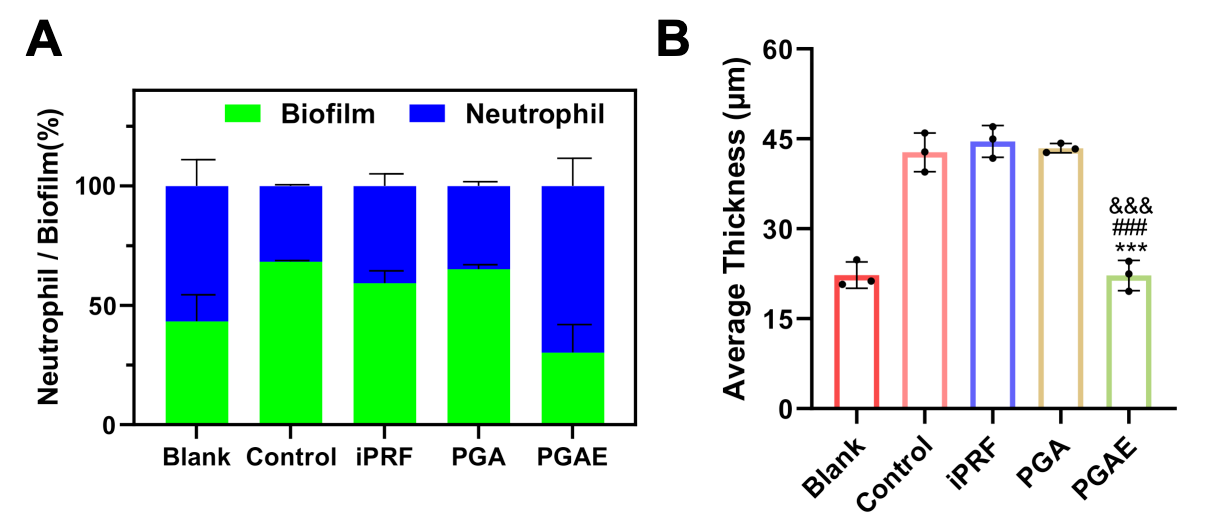


**Figure S13.** Effects of hydrogel-treated neutrophils on multispecies bacterial biofilms. (A) The ratio of neutrophils to bacteria; (B) Quantitative analysis results of biofilm thickness in each group. (Compared with the Control group, "*" means *p* < 0.05, "**" means *p* < 0.01, and "***" means *p* < 0.001; Compared with iPRF group, "#" means *p* < 0.05, "##" means *p* < 0.01 and "###" means *p* < 0.001; Compared with PGA group, "&"means *p* < 0.05, "&&"means *p* < 0.01 and "&&&"means *p* < 0.001.)


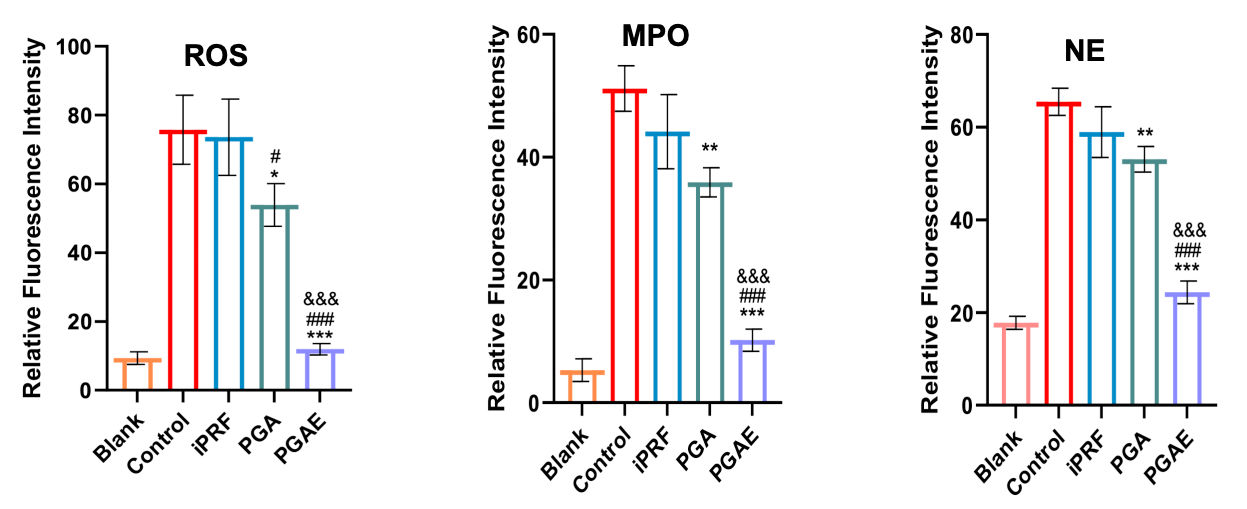


**Figure S14.** Fluorescence intensity analysis of ROS, MPO and NE. (Compared with the Control group, "*" means *p* < 0.05, "**" means *p* < 0.01, and "***" means *p* < 0.001; Compared with iPRF group, "#" means *p* < 0.05, "##" means *p* < 0.01 and "###" means *p* < 0.001; Compared with PGA group, "&"means *p* < 0.05, "&&"means *p* < 0.01 and "&&&"means *p* < 0.001.)


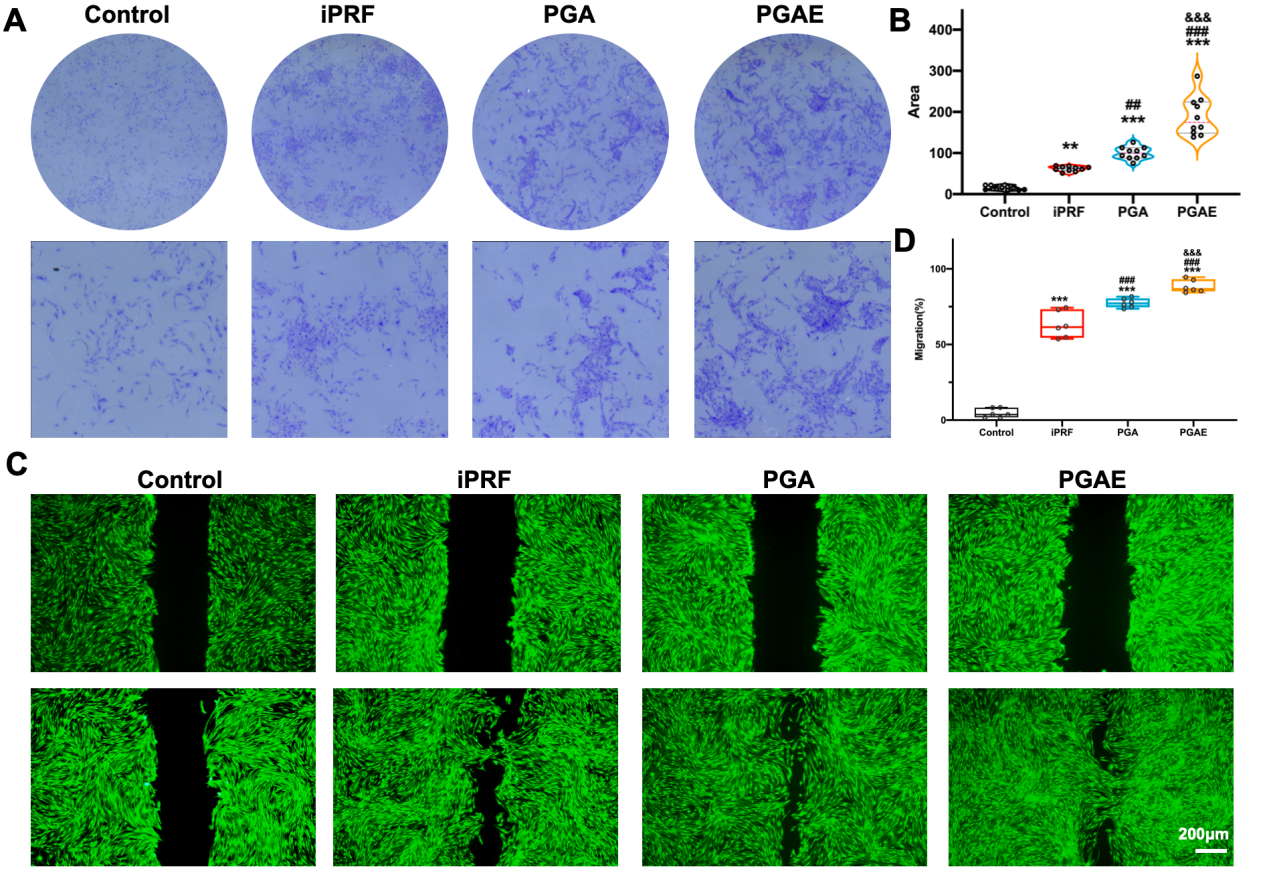


**Figure S15. Effects of biochemical signals released from hydrogels on the proliferation and migration capacities of GFs.** (**A**) Results of the colony formation assay. (**B**) Quantitative analysis of colony area. (**C**) Migration capacity of GFs influenced by different hydrogels. (**D**) Quantitative analysis of cell migration rate. (Compared with the Control group, "*" means *p* < 0.05, "**" means *p* < 0.01, and "***" means *p* < 0.001; Compared with iPRF group, "#" means *p* < 0.05, "##" means *p* < 0.01 and "###" means *p* < 0.001; Compared with PGA group, "&"means *p* < 0.05, "&&"means *p* < 0.01 and "&&&"means *p* < 0.001.)


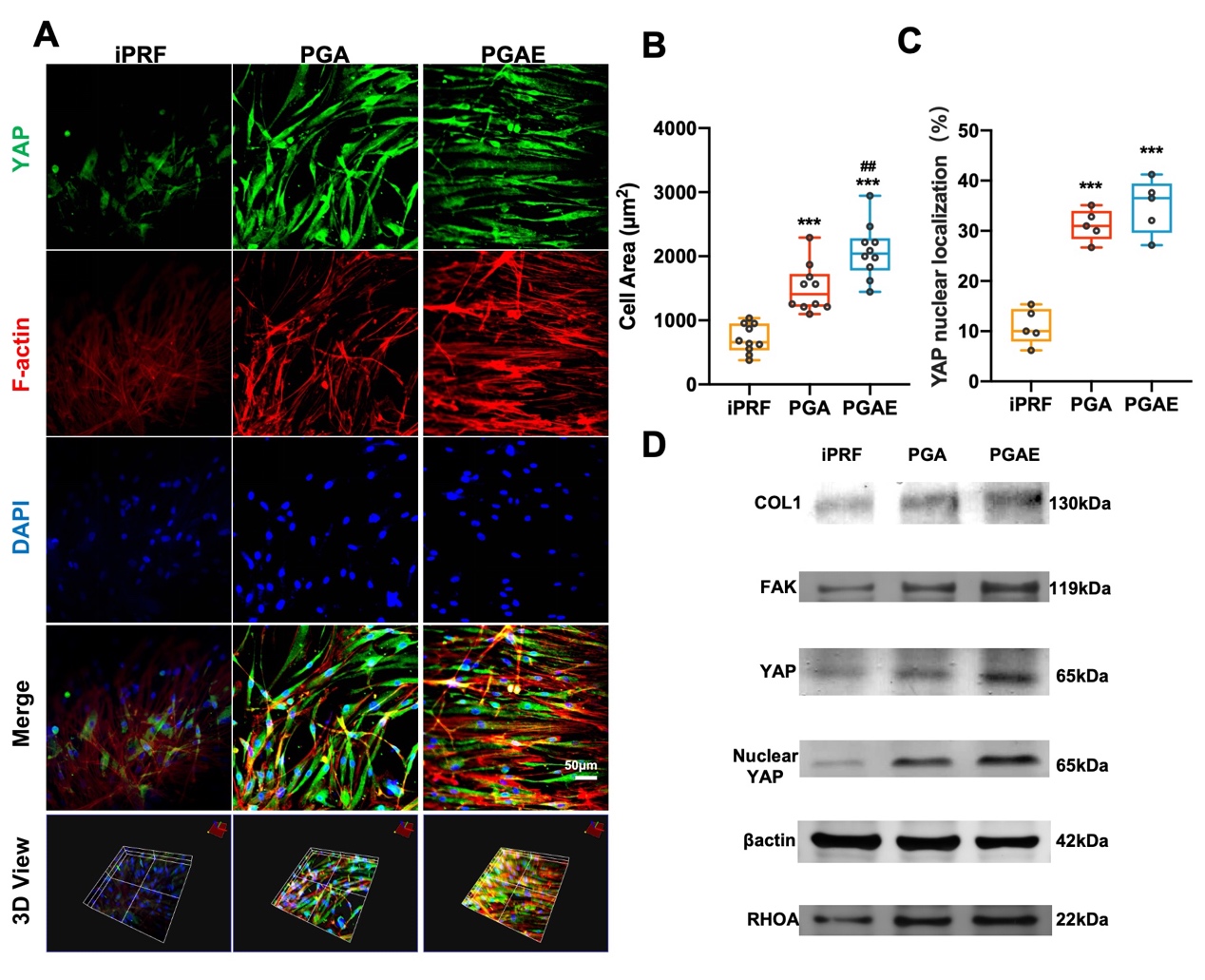


**Figure S16.** Representative western blot bands for FAK, RHOA, total YAP, nuclear YAP, and COL1.


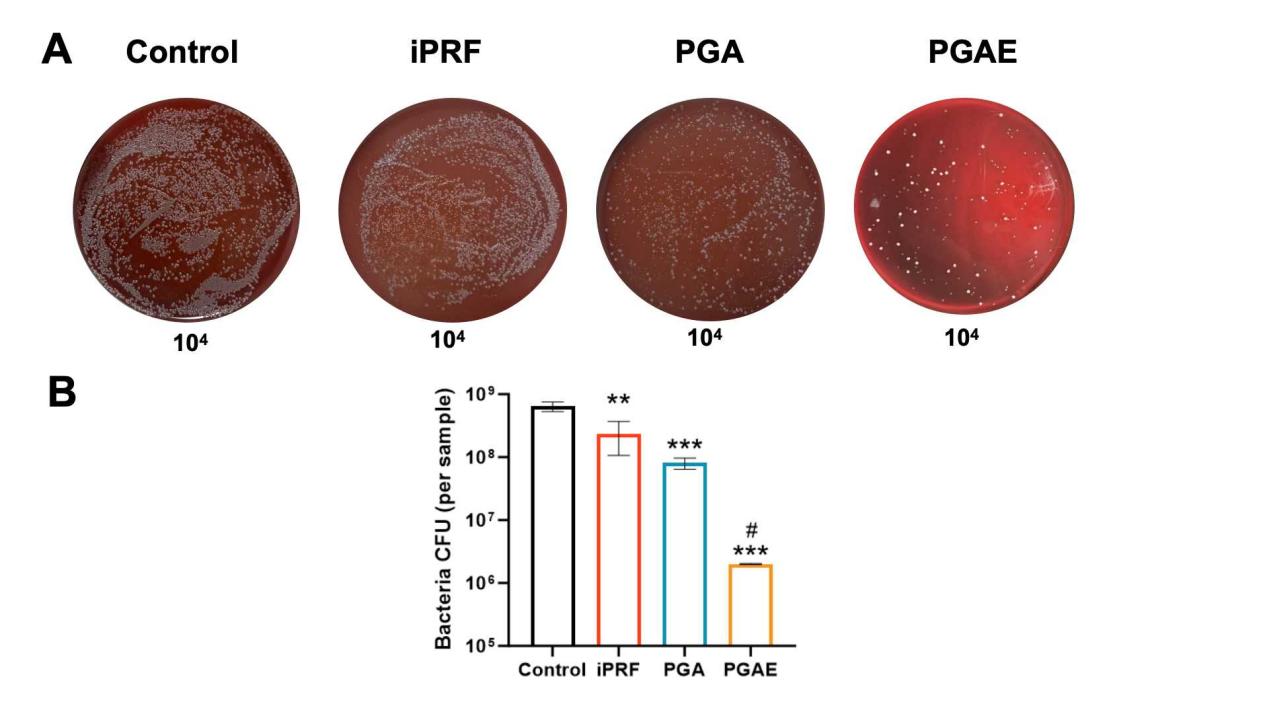


**Figure S17.** Evaluation of in vivo antibacterial properties of different hydrogels (A) Bacterial colonies collected from tissue defects were cultured on agar plates, and the numbers below represent the dilution ratio; (B) CFU counts corresponding to each sample. (Compared with the Control group, " * " means *p* < 0.05, " ** " means *p* < 0.01, "*** " means *p* < 0.001; compared with the iPRF group, " # " means *p* < 0.05.)


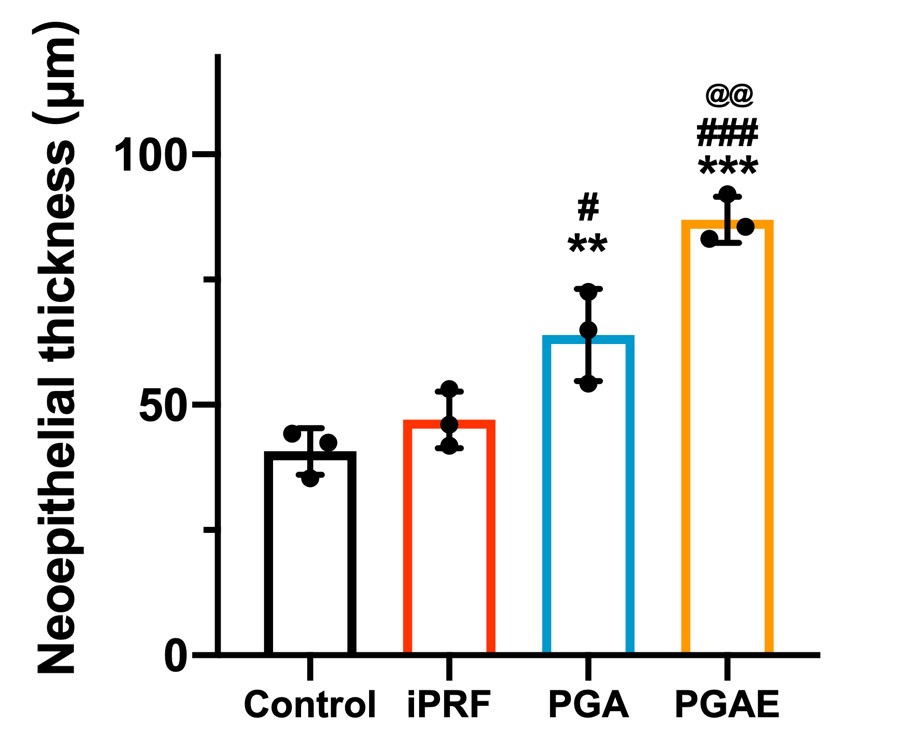


**Figure S18.** Quantitative analysis results of the thickness of new epithelial tissue in each group. (Compared with the Control group, "*" means *p* < 0.05, "**" means *p* < 0.01, and "***" means *p* < 0.001; Compared with iPRF group, "#" means *p* < 0.05, "##" means *p* < 0.01 and "###" means *p* < 0.001; Compared with PGA group, "@"means *p* < 0.05 and "@@"means *p* < 0.01.)


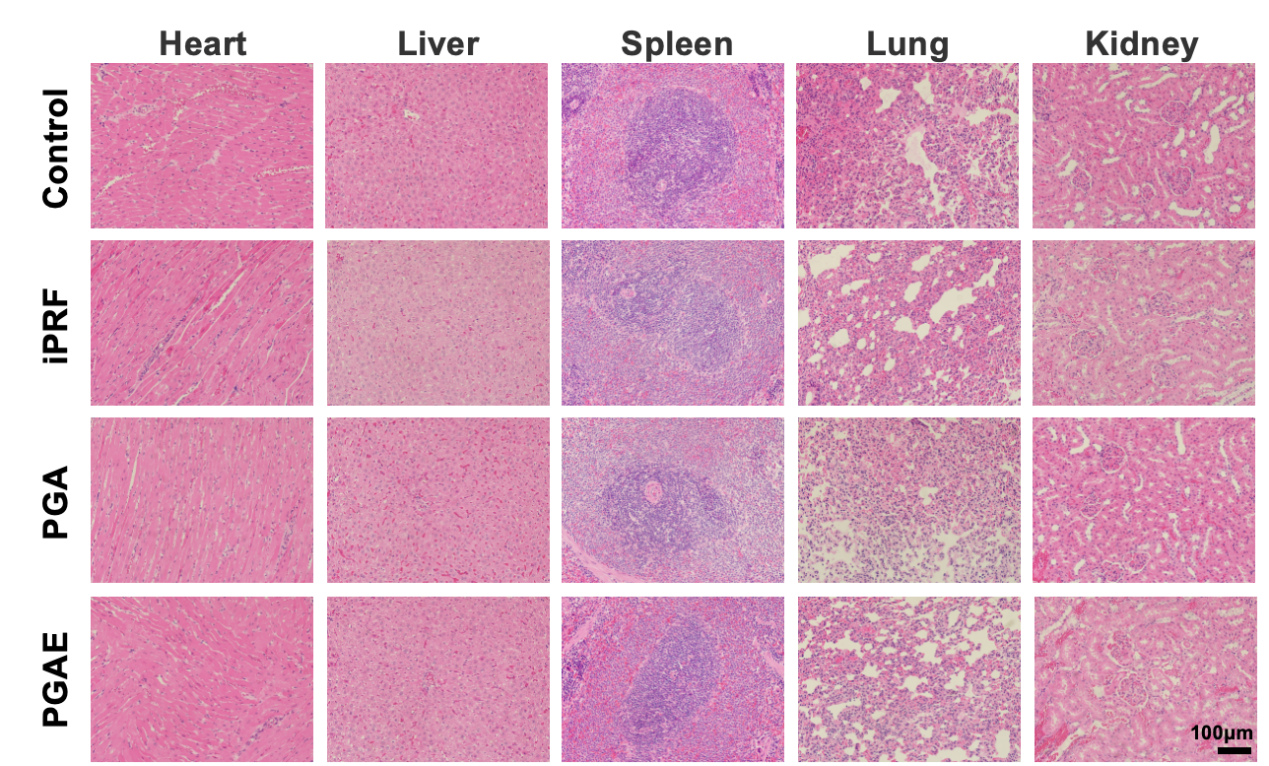


**Figure S19.** HE staining results of major organs in different groups.

**Table S1.** Primer sequences of inflammation-related genes

| Gene name | Forward Primer (from 5’-3’) | Reverse Primer (from 5’-3’) |
| --- | --- | --- |
| *gapdh* | CATCCGTAAAGACCTCTAGCCAAC | ATGGAGCCACCGATCCACA |
| *tnfα* | ACTCCAGGCGGTGCCTATGT | GTGAGGGTCTGGGCCATAGAA |
| *il6* | CCACTTCACAAGTCGGAGGCTTA | CCAGTTTGGTAGCATCCATCATTTC |
| *il10* | ATGCTGCCTGCTCTTACTGACTG | CCCAAGTAACCCTTAAAGTCCTGC |
| *arg* | CATTGGCTTGCGAGACGTAGAC | GCTGAAGGTCTCTTCCATCACC |

**Table S2.** Primer sequences of genes related to mechanotransduction signaling pathways

| Gene name | Forward Primer (from 5’-3’) | Reverse Primer (from 5’-3’) |
| --- | --- | --- |
| GAPDH | GTCTCCTCTGACTTCAACAGCG | ACCACCCTGTTGCTGTAGCCAA |
| FAK | GAATTGGGCGGAAAGAAATCCT | AAGCCTCCATGGCTTGACAC |
| RHOA | AGCCTGTGGAAAGACATGCTT | TCAAACACTGTGGGCACATAC |
| COL1 | GCCCTGCTGGTGCTCG | ACCCTGGGGACCTTCAGAG |
| FN | CCGCCGAATGTAGGACAAGA | CTGTCAGAGTGGCACTGGTA |
